# Grown and thrown: Exploring approaches to estimate food waste in EU countries

**DOI:** 10.1016/j.resconrec.2021.105426

**Published:** 2021-05

**Authors:** Carla Caldeira, Valeria De Laurentiis, Agneta Ghose, Sara Corrado, Serenella Sala

**Affiliations:** a European Commission -Joint Research Centre, Via Enrico Fermi 2749, I-21027, Ispra, VA, Italy; b Department of Planning, Rendsburggade 14, Aalborg University, 9000 Aalborg, Denmark

**Keywords:** Food waste, EU countries, Material Flow Analysis, Waste statistics

## Abstract

National studies on food waste quantification in EU countries present highly discrepant results due to the different quantification approaches adopted. The European Commission has published a delegated act establishing a common methodology and minimum quality requirements for the uniform measurement of food waste generated in Member States. Nevertheless, as EU countries are at different levels of development and implementation of national strategies for food waste quantification, there is a need to develop a harmonized modelling system that enables the estimation of food waste generated by Member States to assess the amounts reported by each country. The aim of this paper is to fulfil this need by presenting two modelling approaches to estimate food waste in EU countries. One approach is based on Material Flow Analysis (MFA) and combines statistical information on the production and trade of food products with food waste coefficients. The other approach is based on the estimation of food waste based on waste statistics. Three EU countries are used to illustrate the two approaches and compare the results obtained thereby. Food waste estimates from waste statistics are generally lower than those obtained using MFA, in particular at the early stages of the food chain. The MFA model presented in this article is the first of its kind developed to estimate food waste across Member States in a consistent way and through time. Crucially, this could support the definition of a baseline and binding targets to reduce food waste across the EU, as announced in the EU Farm to Fork Strategy.

## Highlights

• Two modelling approaches for food waste estimation at country level are presented.
• Three EU countries (Italy, Germany, and Denmark) are used to illustrate the models.
• Discrepancies between the two methods’ results at early stages of the food chain.
• EU Waste statistics are limited in providing an estimation of food waste.
• Mass Flow Analysis can be used to assess food waste estimates for EU countries.

## Graphical abstract

**Figure:**
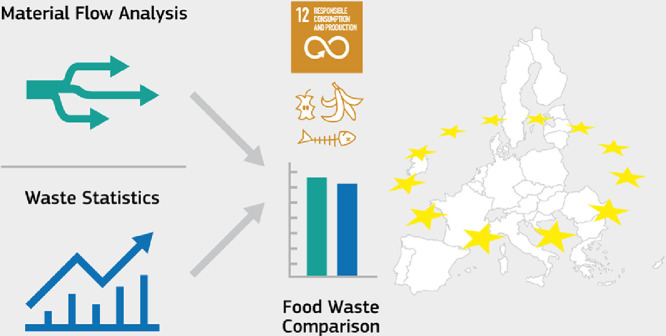

## 1 Introduction

Food waste is responsible for significant economic, environmental, and social impacts (FAO, 2019) and the implementation of strategies for its reduction is imperative. A cornerstone in the fight against food waste was the definition of Sustainable Development Goal (SDG) target 12.3: ‘by 2030 halve per capita global food waste at the retail and consumer levels, and reduce food losses along production and supply chains including post-harvest losses’ (UN, 2015). Since then, the European Commission (EC) has been dedicated to fight food waste and has committed to achieve the SDG 12.3 target, first in the European Circular Economy Action Plan (European Commission, 2015) and by reiterating this objective in the recently published Farm To Fork strategy (European Commission, 2020).

A fundamental step in the fight against food waste is its quantification. The knowledge of the amounts of food wasted at each step of the value chain is key for the design of efficient food waste prevention strategies. Up to now, studies quantifying food waste have been developed using different approaches and data sources, mainly relying on secondary data, resulting in high uncertainties in the existing global food losses and waste database (Xue et al., 2017). Corrado and Sala (2018) analyzed studies developed at global scale and at EU level, showing significant discrepancies in the results, due to different quantification approaches and data sources used. Caldeira et al. (2019a) reached a similar conclusion in a review of the existing studies assessing food waste generation at national level by EU Member States (MSs), stating that methodological differences limited the comparability of the results and the monitoring of food waste generation over time among EU countries.

To contribute to the harmonization of food waste quantification in the EU the EC has published a Delegated Decision (EU) 2019/1597 of 3 May 2019 establishing a common methodology and minimum quality requirements for the uniform measurement of food waste generated in MSs (European Commission, 2019). The delegated act is expected to contribute to reduce the discrepancy in the state of play on food waste quantification among the different MSs by providing a common approach for MSs to develop their studies. While this is foreseen to improve the knowledge of food waste generation across the EU, many MSs are only now starting to develop and implement national strategies for food waste quantification. The first reporting of food waste from MSs, following the guidelines provided in the delegated act, is due by June 2022 for the reference year 2020. Against this backdrop, there is the need to develop a harmonized modelling system, enabling the estimation of food waste generated by MSs and providing a common baseline for countries. The aim of this paper is to fulfil this research need by presenting two modelling approaches to estimate food waste at MS level.

The first modelling approach uses statistics on food production and trade combined with food waste coefficients to estimate food and food waste flows across the food chain. Examples of this approach are the works by Bräutigam et al. (2014), FAO (2011), Porter et al. (2016), Kemna et al. (2017), and Caldeira et al. (2019b). These studies generally rely on FAO food balance sheets (FAO, 2020) as source of data for food supply combined with food waste coefficients taken from different sources. In FAO (2011), food waste coefficients were collected from a range of different sources, such as scientific literature, statistical databases, national authorities, and Non-Governmental Organizations. The sources of the coefficients used are presented in Gustavsson et al. (2013). Bräutigam et al. (2014) adopted FAO's approach, considering the same waste coefficients, except for the ‘postharvest handling and storage’ stage, which were instead directly calculated from data given in the food balance sheets. Porter et al. (2016) used average coefficients (when data were available) to overcome the possible limited representativeness and accuracy of some punctual coefficient reported by FAO (2011). The main difference between the above-mentioned studies is the type of food waste considered (i.e. edible and inedible). FAO (2011) and Gustavsson et al. (2013) have quantified only edible parts of food wasted, whilst the others have accounted for both edible and inedible parts. The studies by Kemna et al. (2017) and Caldeira et al. (2019b) also used food production statistics and waste coefficients but following a material flow analysis (MFA), which is a ‘systematic assessment of the flows and stocks of materials within a system defined in space and time’ (Brunner and Rechberger, 2004) ensuring a closed mass flow analysis from stage to stage.

Using a combination of statistical data sources and scientific literature, which included estimates of food waste coefficients, Kemna et al. (2017) accounted for food waste at EU level. The work developed by Caldeira et al. (2019b) builds on Kemna et al. (2017) by additionally providing a systematized approach to perform food waste accounting at EU level, including a compilation of coefficients that can be used to fill data gaps when quantifying food waste. Other studies that adopted an MFA-based approach were focused on case studies: e.g. Beretta et al. (2013) accounted for food waste in Switzerland; Courtonne et al. (2015) performed an MFA of the cereal supply chain in France and quantified losses at farm and during grain storage, and residues from transformation industries; and Xue et al. (2019) mapped the dry matter mass and energy balance of the meat supply chain in Germany (considering beef, pork, and poultry), including waste.

The second modelling approach uses waste statistics to estimate food waste generated across the food supply chain. Examples of this approach are the works by Monier et al. (2010) and Eurostat (2017). In these studies, food waste is estimated using data from EU waste statistics reported by each MS and collected by Eurostat, following the European Waste Classification for statistical purposes (EWC-Stat) (European Commission, 2010). In this classification, food waste is not reported separately, but is included to different extents in a number of waste categories together with other bio-waste streams. To overcome this issue, Monier et al. (2010) replaced Eurostat figures with national data, where available and of sufficient quality. In Eurostat (2017), data at a higher level of detail than usual (i.e. using waste codes presenting higher granularity) was collected, using the administrative classification List of Waste (LoW), for which a conversion table from the substance oriented classification EWC-Stat exists (European Commission, 2010), classifying each LoW code based on whether it ‘contains’, ‘partly contains’ or ‘does not contain’ food waste. Following such rationale, Eurostat (2017) estimated the food waste contained in the total waste reported in the selected waste codes.

Building on the works of Caldeira et al. (2019b) and of Eurostat (2017), this paper explores and compares methods to estimate food waste at national scale drawing on existing statistical data. The modelling approaches are described in Section 2 and results obtained from their application to three EU countries used as case studies are presented and discussed in Section 3. Finally, conclusions drawn from this work are presented in Section 4.

## 2 Method

Two alternative methodological approaches to estimate food waste at national level are presented in this article. The first builds on the work done by Caldeira et al. (2019b) and is based on MFA (hereafter designated as “MFA approach"). In this approach, statistical information on production and trade of fresh and processed food products is combined with technical coefficients of processed food production and waste coefficients taken from the literature to estimate waste and by-products generated along the food supply chain.

The second approach estimates the amount of food waste based on waste statistics (WS) (hereafter designated as “WS approach”).

**Fig. 1:**
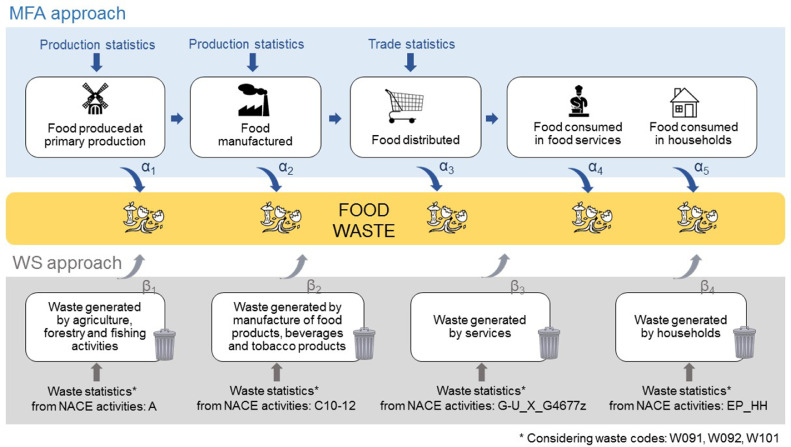
Representation of the two approaches investigated for the estimation of food waste quantities. MFA: material flow analysis, WS: waste statistics.

Fig. 1 illustrates the two approaches, which are described in detail in Sections 2.1 and 2.2, respectively. Both approaches quantify food waste in wet mass.

The models can be used to estimate food waste for all MSs; as an illustrative example, three MSs were chosen to present the results: Italy, Germany, and Denmark. These countries were selected as they present different production and consumption patterns, and due to the availability of country-specific coefficients to calculate the share of food waste in household waste. A comparison between the results of the two approaches is presented for the year 2012. The choice of the reference year is related to the availability of food consumption data (EFSA, 2015), which is used to perform a plausibility check of the output of the MFA model (Section 3.2.1).

### 2.1 Quantification of food waste in EU countries using the material flow analysis approach (MFA)

This approach is based on the model developed by Caldeira et al. (2019b) at EU level. For the scope of this paper, the model was updated to provide results at MS level.

In the original model (Caldeira et al., 2019b), food waste was defined as: ‘food and inedible parts of food removed from the food supply chain’ to be recovered or disposed (including: composted, crops ploughed in/not harvested, anaerobic digestion, bioenergy production, co-generation, incineration, disposal to sewer, landfill or discarded to sea) (FUSIONS, 2014).

Instead, in the current model (version 1.0) the food waste definition has been aligned with the EU food waste definition (see Section 2.2), which does not include crops left in field or ploughed-in and the mortality of animals ready for slaughter in the accounting of food waste. These quantities are nevertheless estimated by the updated model, and are designated as “food losses”.

Food waste amounts are calculated at the different stages of the food chain, i.e. primary production, manufacturing, retail and distribution, and consumption (including food services and households), and are reported in wet mass. The model follows a territorial approach in which food waste embedded in the net imports of raw and manufactured products is not accounted for.

The model uses multiple sources of data, namely:

• FAOSTAT: production of crops, processed crops, livestock, livestock products, trade of crops and livestock products, commodity balance sheets (CBS) (FAO, 2020). The FAO CBS provide data on the quantities of different commodities used for food, feed, seeds, processing, and other non-food related uses (e.g. bioenergy).
• Eurostat: production of crops, livestock products, manufactured food products, trade of crops and livestock products (Eurostat, 2012).
• Industry association (e.g. European Potato Processors' Association): Inputs/outputs of food manufacturing by country and products.
• Scientific literature providing food waste coefficients (i.e. waste as a percentage of the input flow) based on direct measurement (e.g. Beretta et al. (2013)).

The original modelling approach is presented in Caldeira et al. (2019b). A number of refinements and updates were done when deriving the model at MS level and to adjust for different data availability at MS level. The updates mostly concerned the modelling of the primary production and manufacturing stages (e.g. the estimation of food waste and by-products generated at the manufacturing stage of potatoes, fish, olive oil and cereals were significantly revised). Detailed information on the updated model and on the underlying data used (databases used, lists of commodities and products extracted from each, coefficients used and respective data source) is provided in SI. The model was implemented in the programming sofware R (R Core Team, 2017).

At both primary production and processing stages, an estimation is provided of the streams generated that are used for animal feed or other processing purposes, hence classified as by-products, and of those that are wasted.

The amounts of food entering the distribution phase are taken from a range of statistical data sources and reports (e.g. Prodcom for processed food products, Freshfel (2019) for fresh fruit and vegetables), and combined with coefficients of food waste at distribution to estimate food waste. Then, the remaining amount (i.e. food entering the distribution phase minus food waste at this stage) is considered to enter the consumption stage and is split between household and food services consumption. This enables the assessment of food waste generated both at household and food service levels using tailored coefficients. Finally, by subtracting the food waste at consumption from the amount of food entering this stage, the model estimates the amounts of consumed food. Although this is not the main purpose of the model, it represents a crucial output as it enables to perform a plausibility check of the full model, by comparing consumed amounts estimated against those reported by nutritional surveys. Such a comparison, for the three case studies developed, is provided in Section 3.2.1.

### 2.2 Quantification of food waste in EU countries using the Waste Statistics method (WS)

The WS approach quantifies food waste based on waste statistics. For this model, food waste refers to “all food as defined in Article 2 of Regulation (EC) No 178/2002 of the European Parliament and of the Council (European Parliament and Council, 2002) that has become waste” (European Commission, 2018), including both edible and inedible parts of food.

Food waste was quantified for the different stages of the food supply chain for specific NACE (Nomenclature statistique des Activités économiques dans la Communauté Européenne) activities i.e.those expected to generate food waste. The correspondence between the NACE activities considered and the stages of the food supply chain is the following:

• primary production corresponds to NACE activity A: Agriculture, forestry and fishing;
• processing and manufacturing corresponds to NACE activities C10-C12: Manufacture of food products; beverages and tobacco products;
• retail and food services correspond to NACE activities G-U_X_G4677: Services (except wholesale of waste and scrap);
• households correspond to NACE activity EP_HH: Households.

The main underlying data source used are the EU waste statistics, reported by each MS and collected by Eurostat based on EU Commission regulation on waste statistics (No. 2150/2002) (European Commission, 2012). The waste statistics were reported in “Generation of waste by waste category, hazard and NACE Rev. 2 activity” (Eurostat, 2014). Data are provided for all economic sectors (following the NACE classification of activities) and for households.

As the waste statistics do not explicitly report food waste, the following European Waste Catalogue for Statistics (EWC-Stat) codes potentially containing food waste were identified in Eurostat (2017):

• Animal and mixed food waste (EWC-Stat W091);
• Vegetal waste (EWC-Stat W092**)**;
• Household and similar wastes (EWC-Stat W101).

The calculations of the total amount of food waste per country is obtained by means of equation 1.

Equation 1

$$FW_{MS}=\sum_{i}\beta_{091}*W_{091,\;i}+\;\beta_{092}*W_{092,\;i}+\beta_{101}*W_{101,\;i}$$

for $i=NACE\;activities\;A,\;\;C10-C12,\;\;G-U_{X_{G4677}},EP\_HH\;$

Where:

$FW_{\text{MS}}$Total amount of food waste in each MS

$\beta_{091}$Coefficient expressing the share of food waste in waste code W091

$W_{091,\;i}$Waste amount reported under waste code W091 by NACE activity i

$\beta_{092}$Coefficient expressing the share of food waste in waste code W092

$W_{092,\;i}$Waste amount reported under waste code W092 by NACE activity i

$\beta_{101}$Coefficient expressing the share of food waste in waste code W101

$W_{101,\;i}$Waste amount reported under waste code W101 by NACE activity i

In the model, the coefficient expressing the share of food waste in waste code W091 ($\beta_{091}\;$) is equal to 0.92 whilst the coefficient expressing the share of food waste in waste code W092 ($\beta_{092}\;$) is equal to 0.59. A coefficient equal to 0.23 was used as a default coefficient for the share of food waste in waste code W101 (*β_101_*), when no country-specific coefficient was available. This was obtained by multiplying the average share of food waste in mixed municipal waste (equal to 0.25) by the average share of mixed municipal waste in W101 (which also includes other types of waste such as waste from markets and street cleaning residues). These coefficients were obtained from (Eurostat 2020) as share of food waste in the EWC categories based on data reported by MSs. It is important to highlight that these coefficients were determined to be used at EU level and differences in the coefficients are observed at MS level. Country specific coefficients cannot be used due to data confidentiality, nevertheless we assessed the potential variation in the results due to differences in the coefficients using the minimum and maximum values reported by the different MSs.

As mentioned in Section 2.1, this work presents the application of this approach to three countries: Italy, Germany and Denmark. To this end, the calculation was refined by adopting country-specific coefficients expressing the share of food waste in waste code W101 ($\beta_{101})$. These coefficients were collected from literature studies performing waste composition analyses of municipal solid waste conducted in each country.

Therefore, $\beta_{101}$ was assumed to be equal to:

• 0.28 for Italy, based on the composition of municipal waste as reported in the national report of municipal waste (Andreasi Bassi et al., 2017; ISPRA, 2014);
• 0.19 for Germany, based on the municipal waste composition reported by the federal environmental agency (Andreasi Bassi et al., 2017; Jensen et al., 2016);
• 0.41 for Denmark, based on the residual waste collected from 1442 households in three Danish municipalities (Edjabou et al., 2016; Edjabou et al., 2015).

## 3 Results and discussion

This section presents a comparison of the amounts of food waste calculated using the two approaches (Section 3.1) and the underlying food waste estimated for each food group across the food supply chain calculated with the MFA approach (Section 3.2) for the three countries used as case studies. The underlying statistical data used refers to the reference year (2012).

### 3.1 Food waste estimated using the Material Flow Analysis and the Waste Statistics approaches

**Fig. 2:**
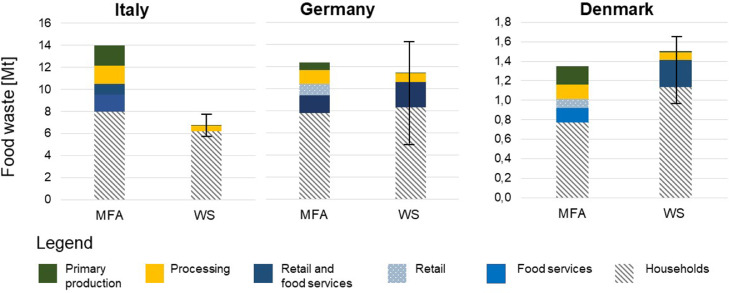
Total amounts of food waste (in million tons of wet mass) for Italy, Germany, and Denmark for the year 2012 calculated using the material flow analysis (MFA) and waste statistics (WS) approaches. The error bars in the WS results represent the variation obtained using the full range of waste coefficients β_091 and β_092 reported by MSs.

The absolute figures of the food waste calculated using the MFA and WS approaches for each country at each stage of the food supply chain are presented in Fig. 2 and Table 1. Although the MFA approach provides distinct results for the retail and food services sector, food waste amounts estimated for these two stages were added together to be compared with the amounts estimated from the WS for NACE activity regarding services (G-U_X_G4677). For the remaining stages of the food supply chain, a direct correspondence was made between primary production and NACE activity A (Agriculture, forestry and fishing), between processing and manufacturing and NACE activity C10-C12 (Manufacture of food products; beverages and tobacco products), and between household and NACE activity EP_HH (households).

**Table 1:** Total food waste (in million tons of wet mass) estimated using the material flow analysis (MFA) and the waste statistics (WS) approaches. Δ refers to the difference in the results of the WS (values obtained with EU-average coefficients) relatively to the MFA approach. Values in brackets refer to food losses amounts.

Country	Approach	Primary production (Mt)	Δ (%)	Processing and Manufacturing (Mt)	Δ (%)	Retail and Food Services (Mt)	Δ (%)	Households (Mt)	Δ (%)
Italy	MFA	1.81 (1.96)	-98	1.69	-71	2.51	-99	7.98	-22
	WS	0.03		0.49		0.03		6.19	
Germany	MFA	0.65 (2.45)	-87	1.30	-40	2.63	-13	7.79	7
	WS	0.08		0.78		2.30		8.32	
Denmark	MFA	0.18 (0.28)	-94	0.15	-48	0.24	17	0.77	47
	WS	0.01		0.08		0.28		1.13	

Table 1 presents the relative difference (Δ) between the results obtained with the WS approach and those obtained with the MFA approach, showing that the amounts reported by the MFA are generally higher than the ones obtained with the WS approach. The largest discrepancies between the results obtained with the two approaches are observed at primary production. This is observed for all the countries, as the WS approach yielded results between 87% and 98% lower than those obtained with the MFA approach. This trend is also observed for the processing stage, although the difference is smaller: the values obtained with the WS approach are 71%, 40%, and 48% lower than the values obtained with the MFA approach, for Italy, Germany, and Denmark, respectively.

The significant difference between the results of the two approaches at primary production can be explained considering that part of the waste flows generated at primary production are disposed of on-site, e.g. via composting, anaerobic digestion. Therefore, none of these quantities are reported in the waste statistics.

In the MFA model, an attempt has been made to estimate the relative share of waste and by-products generated at the processing stage for the different food groups, and when possible, considering the different products modelled within each group (the reader is referred to the SI for more details). However, finding country-specific data sources to model this stage proved particularly challenging, and, therefore, in most cases, average EU coefficients are used for all countries. Consequently, the model might not be representative of the specific countries or specific products. This could entail that the share of food waste generated by the model is overestimated (and the share of by-products underestimated), which might explain the higher values obtained with the MFA compared to the WS at this stage. Additionally, it is quite common that the treatment of residues generated from food manufacturing takes place on site (e.g. incineration of residues for energy production, anaerobic digestion) and therefore would not be captured by waste statistics. Hence, also in this case, the waste reported in the statistics is expected to be lower than the waste estimated by the model, which includes all streams that become waste (both on- and off-site).

At retail and distribution, the differences in the results obtained with the two approaches are generally less significant. The WS method provides a 13% lower value than the MFA for Germany and a 17% higher value for Denmark. However, in the case of Italy, the value reported by the WS is 99% lower than the value obtained with the MFA approach. Contributing to this difference is the fact that, for Italy, the amount reported under waste code W101, for all NACE activities except for EP_HH, is equal to zero. At retail and distribution, the MFA approach estimates the amount of food waste potentially generated by the three countries based on the amounts of food products entering this stage and on average (i.e. non country-specific) food waste coefficients extracted from the literature. However, in some contexts part of such food might be collected for animal feed production or for value added products (e.g. rendering of meat to produce fats) (Albizzati et al., 2019; Mena et al., 2014) and therefore, one should consider the possibility of overestimated amounts obtained with the MFA approach.

Irrespectively of the approach taken, the dominant role of food waste at consumption is observable using both approaches (Fig. 2). The differences between the results of the WS and MFA approaches are less evident at this stage, particularly for Italy where the former yields a 22% lower value than the latter. For Germany and Denmark, values obtained by the WS are higher than those obtained by the MFA (7% and 47%, respectively). A possible reason to explain this might be the fact that coefficients β_91_ and β_92_ are not country specific and β_101_ might not be representative of the entire country. At household level, the MFA approach estimates food waste generated regardless its destination, including food waste disposed of via the sink, home composted or fed to pets. In some countries, these destinations resulted to be a significant component of household food waste. For instance, according to (Flemish Food Supply Chain Platform for Food Loss 2018), 45% of food waste generated in Flemish households between 2016 and 2018 was either composted or fed to pets. In another study, Kranert et al. (2012) estimated the fraction of uncollected food waste (including food that is home composted, disposed of via the sewer, and fed to pets) in Germany to be 24% for the households and 11 % for food services. These waste amounts are not captured by waste statistics, which could explain the lower results obtained for Italy with the WS approach compared to the MFA approach.

Furthermore, limitations of the WS approach are related to the assumptions needed to estimate food waste amounts from the waste reported in the waste statistics for waste codes W091, W092, and W101, which might affect the accuracy of the results provided. The estimation of the first two coefficients was done using data provided by MSs on a voluntary basis. Such data is very often estimated and there might be some discrepancy between the codes used in the countries and the EWC-stat code, which can, in combination with other factors, influence the results as illustrated by the error bars in the WS results in Fig. 2.

Country-specific coefficients were used to estimate the share of food waste in waste code W101 as explained in Section 2.2. However, such share can vary significantly depending on whether the waste collection system includes source segregation of food waste or not, causing variations across and within countries (Edjabou et al., 2015). Additionally, the food waste contribution to household waste varies between urban and rural areas (where it is more likely that food waste will be home-composted or fed to animals) (Lebersorger and Schneider, 2011). A unique and static coefficient at national level is therefore not able to capture such variations and might result in an overall under/over estimation of the food waste.

Another important aspect to consider that might explain differences obtained with the two approaches is the influence of the water content on the food waste weight. Depending on the time that has passed between the disposal of the waste and its weighting, the temperature to which it is exposed, and the type of collection system in place (e.g. different types of bins) a significant variation in the food waste weight might occur due to water evaporation. For example, in a study developed by Martin (2010), an 8% weight loss over 1 week (in the months May-July) with aerated bins systems was observed.

The estimation of food waste on a macro scale is not trivial. There are disadvantages with respect to data availability and related uncertainty. Both approaches use data from national statistics published by FAO or Eurostat, affected by a degree of uncertainty. It is difficult to determine the level of uncertainty as the data is reported from multiple sources and it is inherently of varying quality.

According to (European Commission (2016)), the EU waste statistics suffer from both coverage errors and differences in data coverage across MSs. Coverage errors are mostly due to: differences in the application of the waste definition; different methodological approaches and priorities in national waste management; and sector-specific coverage problems. One of the reasons for differences in data coverage is how the distinction between waste and by-products is made for the economic activities ‘agriculture, forestry and fishing’ and ‘manufacturing’, in particular for the waste category ‘animal and mixed food waste’, where food waste is generated alongside other types of waste. Due to common definitions and classifications, the comparability of data across countries is fairly high for most sectors and waste types. However, some problems in comparing data across countries still arise due to the differences in coverage described. Furthermore, most countries have their own waste codes and there are existing discrepancies on how these codes are translated to Eurostat codes (Eurostat, 2014). This leads to errors in reporting waste generated in selected categories.

A major difference between the two methods is related to how food waste is defined. In the MFA approach, food waste is estimated using a standard definition specifying the system boundary. This is not the case for food waste estimated from waste statistics, where food waste is not reported as a separate category and it was assumed to be a portion of the waste reported in specific categories that contain food waste.

In addition, there is a temporal disparity in the availability of data, as waste statistics are reported every two years by Eurostat (Eurostat, 2017), while the MFA approach requires data from multiple sources which are not updated as frequently (for example part of the FAO statistics used are available with a three-year gap relatively to the current year).

Benefits of the MFA approach are that: (i) it includes all food waste generated regardless of its destination, (ii) it provides an estimation of the consumed amounts of different food products, enabling to perform a plausibility check of the results (Section 3.2.1), and (iii) it provides an estimation of food waste at food group level, enabling the identification of hotspots (i.e. the most prominent types of food wasted in a specific country). The last aspect is particularly relevant, as by providing a detailed picture of the quantities and typologies of food wasted across the supply chain, it enables to assess the environmental burden of such waste, thus supporting the design of tailored food waste prevention initiatives, tackling not only the food groups that are mostly wasted, but also those causing higher impacts (De Laurentiis et al., 2020).

However, this approach presents some drawbacks. One is that it largely relies on the use of food waste coefficients, which are available for few countries and may not be representative enough due to limited sample for data collection and biases in the methods used to collect data (e.g. surveys very often lead to an underestimation of food waste generation). Furthermore, due to the inherent cost of collecting such data, it is very rare that this is done periodically, enabling to establish a temporal trend (e.g. a reduction in food waste generation). This means that a coefficient collected in one MS might be used to model household food waste generation in the remaining MSs, and that such coefficients are static (if not updated regularly), meaning that the model would not able to capture changes in waste generation patterns caused by e.g. awareness campaigns or national programs to tackle food waste. Furthermore, this approach is affected by gaps in statistical data caused by e.g. confidentiality issues. This aspect is particularly relevant when the model is applied at MS level, as aggregated values at EU level are generally provided even when they are not provided at national level for all MSs. Consequently, for countries not reporting the production or trade of certain products, the amounts of food entering the distribution phase might be underestimated (if either the production or imports are not reported) or overestimated (if exports are not reported) and subsequently, the accuracy of food waste estimation in the following stages is affected.

#### 3.1.1 Comparison with other studies

Very few studies can be found in the literature quantifying food waste in EU MSs. Table 2 presents a comparison between food waste reported in the literature and the results of this study. It should be noted that, in some cases, the studies report results referring to different years than 2012.

**Table 2:** Comparison of the food waste (in Mt) obtained in this study using the MFA and the WS with results from the literature.

**Country**	**Reference**	**Year**	**Primary Prod.**	**Processing & Manuf.**	**Retail & Dist.**	**Consumption**		**Total food chain**
						Food services	Households	
**Italy**	Buchner et al. (2012)	2005-2010	17.70	1.89	0.26	–	–	**–**
	MFA	2012	1.81	1.69	0.96	1.55	7.98	**14.00**
	WS	2012	0.03	0.49	0.03		6.19	**6.75**

**Germany**	Eberle and Fels (2016)	2010	–	–	–	1.93$	6.22	**–**
	Kranert et al. (2012)	2011	–	1.85	0.55	1.90	6.67	**10,97**+
	Schmidt et al. (2019)	2015	1.36	2.17	0.49	1.69	6.14	**11.86**
	MFA	2012	0.65	1.30	1.03	1.59	7.79	**12.37**
	WS	2012	0.08	0.78	2.30		8.32	**11.48**

**Denmark**	Edjabou et al. (2016)	2011/2012	–	–	–	–	0.48	**–**
	Tonini et al. (2017)	2002-2016	0.10&	0.13&	0.16&	0.06&	0.26&	**0.72**&
	Franke et al. (2016)	2010-2013	0.12	–	–	–	–	**–**
	MFA	2012	0.18	0.15	0.09	0.15	0.77	**1.34**
	WS	2012	0.01	0.08	0.28		1.13	**1.50**

$ values calculated from the per capita value

+ values calculated as the sum of the stages considered in the study and not including the entire food chain

& only edible parts of food

Regarding the total amount of food waste reported in the different studies, for Germany, the total values reported in Kranert et al. (2012) and Schmidt et al. (2019) are similar to the values determined by the MFA and WS approaches. However, the figure reported by Kranert et al. (2012) does not includes food waste generated at primary production. For Denmark, the figure reported by Tonini et al. (2017) is lower (about half) than the ones obtained in this study with both approaches. This is to be expected, as the estimation by Tonini et al. (2017) included only the edible parts of food waste, while the amounts obtained in our study include both edible and inedible parts of food. For Italy, no study was available reporting data for the entire food supply chain.

The values of food waste estimated with the MFA approach at the different stages of the supply chain are generally higher than the values reported in the studies from the literature. A major discrepancy is observed between the values reported in Buchner et al. (2012) for the food waste at primary production for Italy (17.7 Mt) and the values reported by the MFA (1.81 Mt) and the WS (0.03 Mt) approaches. However, the first value refers to the share of the agricultural production remaining in the field that was calculated by estimating the difference between the total production and the harvested production. The amount of agricultural production left on field was estimated through the MFA approach, yielding for Italy a value of 1.96 Mt (Table 1). As pointed out by Caldeira et al. (2019b), studies on the amounts of food that is left in fields are limited which makes it difficult to assess which of the two values is closer to reality. Exceptions are the studies done by Schneider et al. (2019) that accounted for food losses in potatoes in Austria and Germany or Johnson et al. (2018) that quantified food losses for six vegetable crops in a farm in the US. The former study revealed that the share of potatoes left in field ranged between 0.2 and 4.9% of the potential yield, while the latter reported an average amount of vegetables left in field equal to 57% of the marketable yield (considering only edible and marketable crops). In this current study, the share of food losses for potatoes is 8.4% of the potential yield whilst for vegetables this share ranges between 0 and 24%.

### 3.2 Country food waste generation profile

The MFA approach allows to derive food waste generated at food group level. Table 3 shows the food waste generated at the primary production and processing and manufacturing stages for each food group for Denmark, Germany, and Italy.

**Table 3:** Food waste estimates (using the MFA approach) at primary production and manufacturing for Denmark, Germany, and Italy (2012). Absolut values (in kt) obtained in this study using the MFA.

	**Food waste at primary production (kt)**			**Food waste at manufacturing (kt)**		
	**Denmark**	**Germany**	**Italy**	**Denmark**	**Germany**	**Italy**
**Meat**	0.00	0.00	0.00	20.13	186.69	143.55
**Fish**	136.50	58.06	66.46	27.89	80.37	37.75
**Dairy**	14.90	89.16	33.45	43.63	218.16	139.81
**Eggs**	1.41	14.42	14.76	1.35	16.90	13.59
**Cereals**	2.06	45.06	18.99	16.42	212.97	44.71
**Fruits**	2.02	99.25	811.30	7.81	135.46	679.84
**Vegetables**	26.61	332.97	868.17	24.05	270.29	570.32
**Potatoes**	0.00	0.00	0.00	9.45	192.46	10.97
**Sugarbeets**	0.00	0.00	0.00	0.00	0.00	0.00
**Oil crops**	1.24	15.18	0.69	0.00	0.01	59.23
**Total**	**184.74**	**654.10**	**1813.81**	148.73	1313.11	**1699.78**

The food groups presenting the largest amount of food waste at primary production are fish for Denmark, and vegetables for Germany and Italy. This is partly influenced by the food waste coefficients used at primary production that are significantly higher for these three food groups compared to the remaining ones, but also by the specific production profile of each country. For instance in the case of Denmark, the production of fruit and vegetables is relatively small compared to the production in Germany and Italy. Instead, fish production is larger, which explains the higher amount of food waste generated by this food group.

Similarly to what is observed for the primary production, Italy presents larger amounts of food waste at manufacturing than the remaining two countries, mostly deriving from fruit and vegetables.

**Fig. 3:**
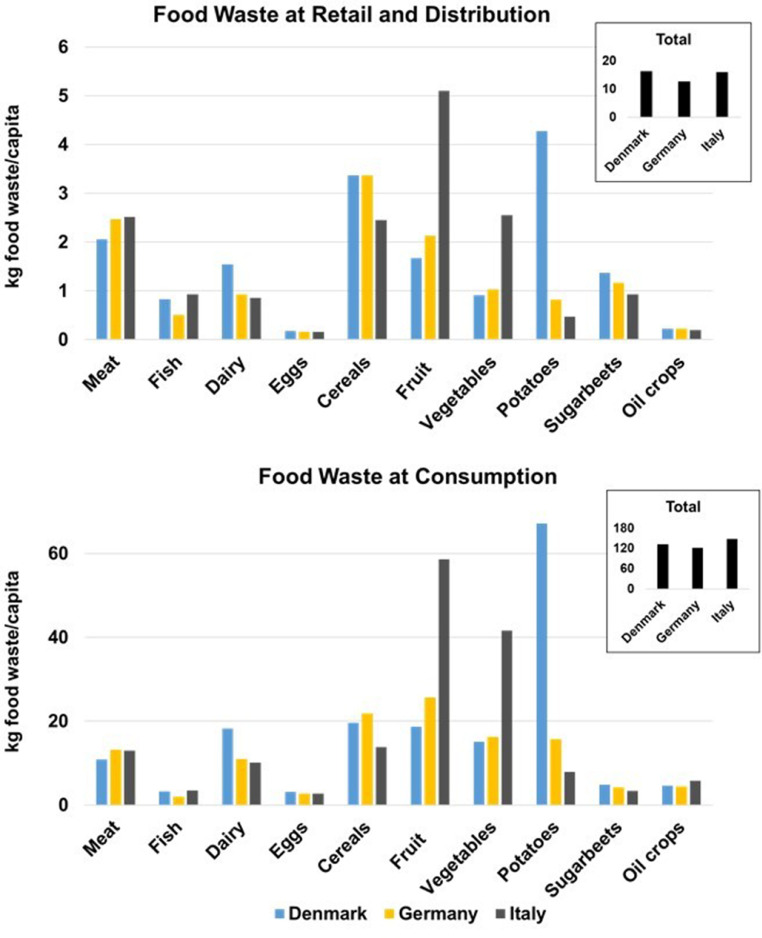
Comparison of the food waste estimated (using the MFA approach) for different food groups at retail and distribution and consumption stages for Denmark, Germany, and Italy (2012). The small graph in the upper-right corner of each graph depicts the sum of the food waste per capita generated in each food group.

To enable a better comparison of food waste calculated for retail and consumption stages across the three countries, per capita results were calculated (Fig. 3), as both stages are closely linked to the quantity of food consumed by the domestic population. The same could not be done for the previous stages of the supply chain as a small country (population wise) might produce large quantities of food for export purposes, or large countries might rely largely on imports.

The waste profile of the different countries (and also the amount of food waste per capita generated) at distribution and consumption is similar (Fig. 3). The highest discrepancies are observed for the fruit and vegetable food groups, where waste generated in Italy is significantly higher than the amounts estimated for Denmark and Germany, and for the potato food group, as waste generated in Denmark resulted significantly higher than amounts generated in Italy and Denmark. Discrepancies for this food group are expected due to assumptions made in the modelling of potatoes at the processing stage caused by numerous gaps in statistical data (for more details the reader is referred to the SI).

#### 3.2.1 Comparison with consumption data

**Fig. 4:**
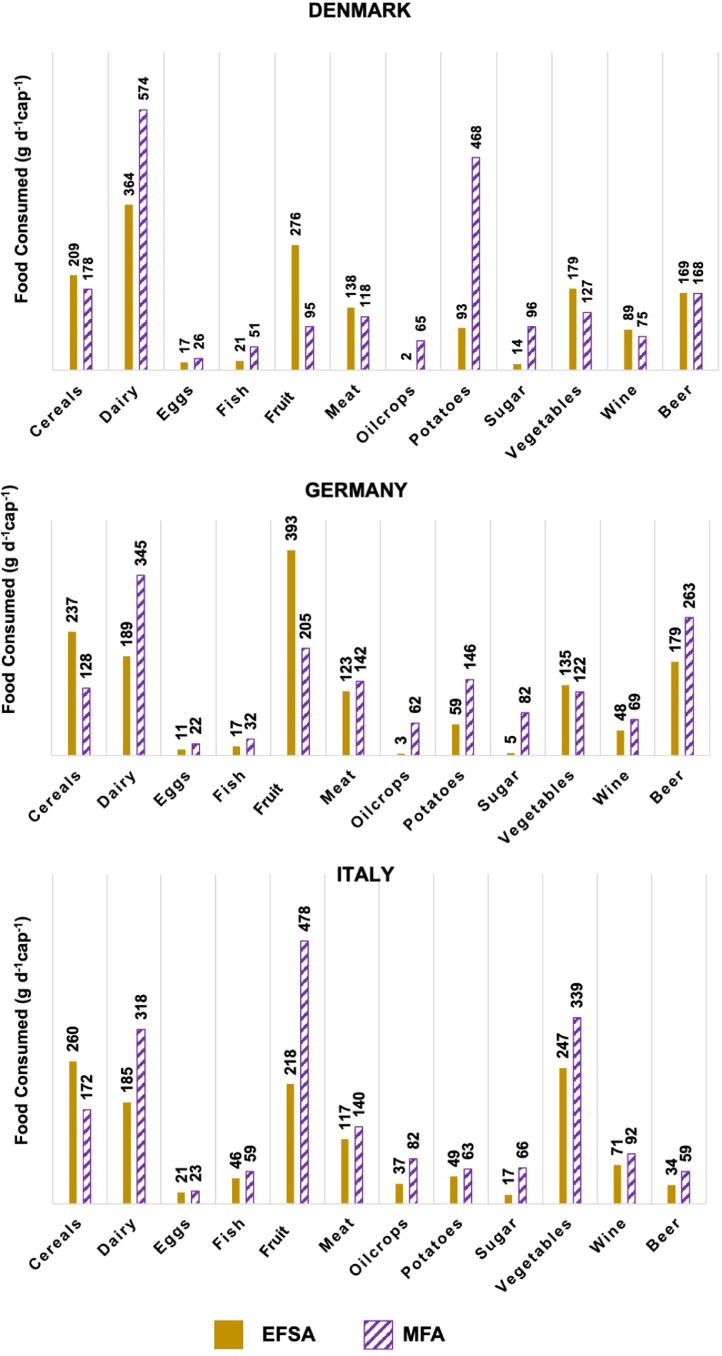
Comparison of the amounts of food consumed (grams per day per capita) for each food group. Data was obtained from EFSA (EFSA, 2015) and from the material flow analysis (MFA) approach.

Aside the amount of food waste and by-products generated along the food supply chain, the MFA approach provides as additional output the amount of food consumed. This was compared with the average food consumption data reported in EFSA (2015) to perform a plausibility check of the output of the MFA model. The rationale followed is that, as the consumed amounts are an output of the model, if those cannot be justified, this might be caused by errors in the model. Fig. 4 compares the amounts of food consumed (in grams per day per capita) obtained with the MFA model with those provided by EFSA. For this comparison an additional breakdown was done, having beer and wine separated from the categories cereals and fruit.

In general, the amounts of food consumed obtained with the MFA approach are higher than the ones reported by EFSA. The food groups for which the food consumed reported in EFSA is higher than the consumed amounts estimated with the MFA approach are cereals for all the countries and fruit for Denmark and Germany. As pointed out in previous work of the authors (Caldeira et al. 2019b), the amounts reported in EFSA might be underestimated as they are based on consumer surveys. According to Leclercq et al. (2009), data collected using this method might be underestimated because people tend to underestimate their food intake.

Additionally, another issue with this comparison is that certain products, such as sugar or vegetable oil, are incorporated into processed food products at the processing stage. As this is not captured by the MFA approach, this model overestimates the amount of e.g. sugar or vegetable oils eaten as such, whilst, in the food consumption surveys on which EFSA data is based, these products would not result as they would be “hidden” under the consumption of processed food products. This makes the estimation of e.g. sugar and fats consumed very difficult, as consumers are rarely aware of the content of the processed products they eat. Therefore, the comparison between the eaten amounts according to the MFA approach and to the food consumption statistics can provide useful insights and enable a plausibility check of the results of the MFA approach only for certain food groups (those that are mostly consumed as such and not as part of other food items, such as vegetables).

## 4 Conclusions

This paper presents two methodological approaches to estimate food waste in EU countries. One approach is based on material flow analysis (MFA) and combines statistical information on the production and trade of food products with food waste coefficients. The other approach estimates food waste based on waste statistics (WS). The two approaches are illustrated by means of case studies based on three EU countries (Italy, Germany, and Denmark), and the results obtained are compared. The added value of performing such a comparison is that it enables to identify potential anomalies as the two approaches rely on different data sources.

Food waste estimates obtained with the MFA approach are generally higher than those obtained using the WS approach. These differences are more significant for early stages of the food chain i.e. primary production and food processing. Such discrepancies are very likely caused by an underreporting of waste collected by waste statistics, as waste flows generated at these stages can be treated on site (e.g. incineration of residues for energy production, anaerobic digestion) and might, therefore, not be reported. Other issues that affect food waste estimates based on waste statistics that can explain the differences obtained are: the influence of the water content on the food waste weight, the fact that countries have their own waste codes and there might be discrepancies between national classification systems and the one adopted by Eurostat, and the uncertainty associated with statistical data used, as such data is reported from multiple sources and it is inherently of varying quality. The latter issue also influences the MFA model as this as well uses statistical data. Nevertheless, this model presents a comprehensive picture of the food system, providing a breakdown of food waste estimates per stage of the food supply chain and per food group that allows the identification of critical food groups and stages. This is particularly relevant, as it can support the design of tailored food waste prevention actions, after combining its findings with environmental considerations to ensure that the food groups presenting highest embedded impacts are given priority. Although country-specific coefficients should be collected to improve the robustness of the MFA approach, the model developed has the potential to be used to assess the data on food waste that will be reported by MSs in the coming years. Crucially, this could support the definition of a baseline and binding targets to reduce food waste across the EU as announced in the Farm to Fork Strategy.

## CRediT authorship contribution statement

**Carla Caldeira:** Writing - original draft, Writing - review & editing, Formal analysis, Methodology, Investigation, Visualization. **Valeria De Laurentiis:** Formal analysis, Methodology, Investigation, Writing - review & editing. **Agneta Ghose:** Investigation, Writing - review & editing. **Sara Corrado:** Methodology, Writing - review & editing. **Serenella Sala:** Conceptualization, Methodology, Validation, Writing - review & editing.

## Declaration of Competing Interest

The authors declare that they have no known competing financial interests or personal relationships that could have appeared to influence the work reported in this paper.

## Appendix B Supplementary materials

Image, application 1

Image, application 2

Image, application 3
