# Supplementary material for: Grown and thrown: Exploring approaches to estimate food waste in EU countries
Source: Resour Conserv Recycl. 2021 May;168:105426. doi: 10.1016/j.resconrec.2021.105426 (PMC7962091; doi:10.1016/j.resconrec.2021.105426)
Supplement: Supplementary file 1 [file mmc1.docx]

**Supplementary Information**

**Grown and Thrown: Exploring approaches to estimate food waste in EU countries**

Carla Caldeira^1^, Valeria De Laurentiis^1^, Agneta Ghose^2^, Sara Corrado^1^, Serenella Sala^1*^

^1^European Commission -Joint Research Centre, Via Enrico Fermi 2749, I-21027, Ispra, VA, Italy

^2^Department of Planning, Rendsburggade 14, Aalborg University, 9000 Aalborg, Denmark

^∗^Corresponding Author; E-mail: [serenella.sala@ec.europa.eu](mailto:serenella.sala@ec.europa.eu)

**Contents**

[1 Detailed methodology of the material flow analysis 2](#_Toc59092407)

[1.1 Primary production (PP) 6](#_Toc59092408)

[1.1.1 Plant based food groups: Sugar beet, Oilcrops, Fruit, Vegetables, Potatoes, Cereals, Oilcrops 7](#_Toc59092409)

[1.1.2 Animal based food groups: Dairy, Eggs, Meat, and Fish 12](#_Toc59092410)

[1.2 Processing and manufacturing (P&M) 14](#_Toc59092411)

[1.2.1 Sugar beet 15](#_Toc59092412)

[1.2.2 Oilcrops 16](#_Toc59092413)

[1.2.3 Potatoes 18](#_Toc59092414)

[1.2.4 Fruit & Vegetables 19](#_Toc59092415)

[1.2.5 Cereals 21](#_Toc59092416)

[1.2.6 Dairy 26](#_Toc59092417)

[1.2.7 Eggs 27](#_Toc59092418)

[1.2.8 Meat 28](#_Toc59092419)

[1.2.9 Fish 31](#_Toc59092420)

[1.3 Retail and distribution (R&D), consumption in household, and consumption in food services 32](#_Toc59092421)

[Annexes 34](#_Toc59092422)

[References 35](#_Toc59092423)

1. Detailed methodology of the material flow analysis

This document provides a detailed description of the underlying methodology of the updated model (version 1.0). For clarity, the methodology is presented differentiating each stage of the food supply chain, and, when necessary, distinguishing between food groups. An overview of the model developed is provided in Figure 1. This representation is valid for all food groups except for meat and fish, which had to be modelled differently due to data availability, as illustrated in Figure 2 and Figure 3. A detailed explanation of the differences between the modelling of meat and fish and the other food groups are provided in subsections 1.1.2, 1.2.8 and 1.2.9.

The aim of this section is to provide all information needed to replicate the model. To this end, the reader is often referred to further information stored in Annex 1 and Annex 2.

Annex 1 contains the full list of coefficients (technical coefficients and food waste coefficients) used by the model with their respective data source. In section 1.2 schemes are provided to illustrate the modelling of the processing stage, where the coefficients used are identified with coefficient codes that are used in the tables of Annex 1.

The tables providing the coefficients used are built to enable the future addition of country-specific coefficients, in case of more studies becoming available. Currently, the following criteria is adopted to select which coefficient to use for a specific country (country A):

- if available, the coefficient from country A is used, otherwise
- if available, a coefficient valid for the EU, otherwise
- if available, a coefficient calculated as the average of the coefficients from other countries (B, C, etc.), otherwise
- a proxy coefficient from a study conducted in a non-EU country or from expert estimation.

Annex 2 contains the full list of databases used, and for each the items extracted with their name (e.g. barley), code in the respective data source (e.g. 2513), and the elements extracted (e.g. production, imports). In addition, when alternative data sources are used by the model (i.e. a second data source is used in case of missing data in the first data source), the correspondence between the product codes from the two data sources are provided in Annex 2.


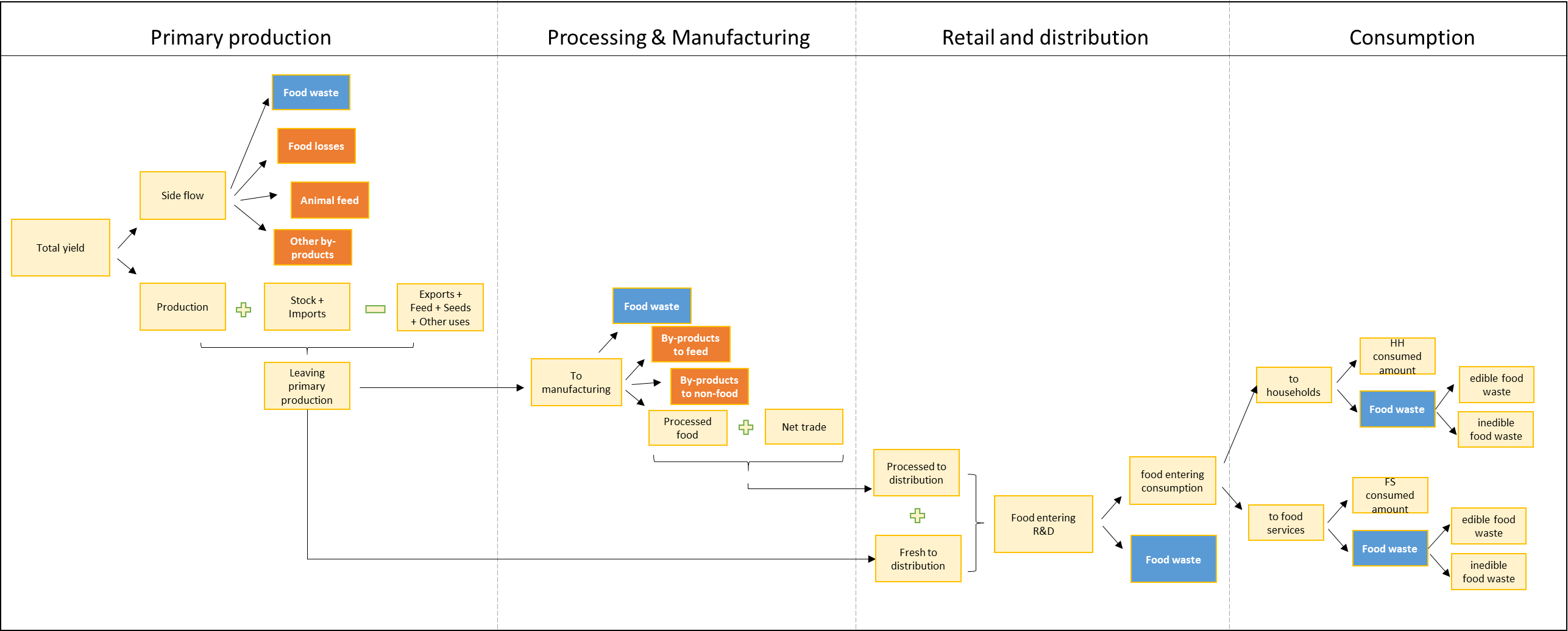


Figure 1. Conceptual scheme of the model developed (v1.0), valid for all food groups except for meat and fish food groups. Trade data at primary production taken from FAO trade and referring to primary commodities only. All items expressed in their real weight.


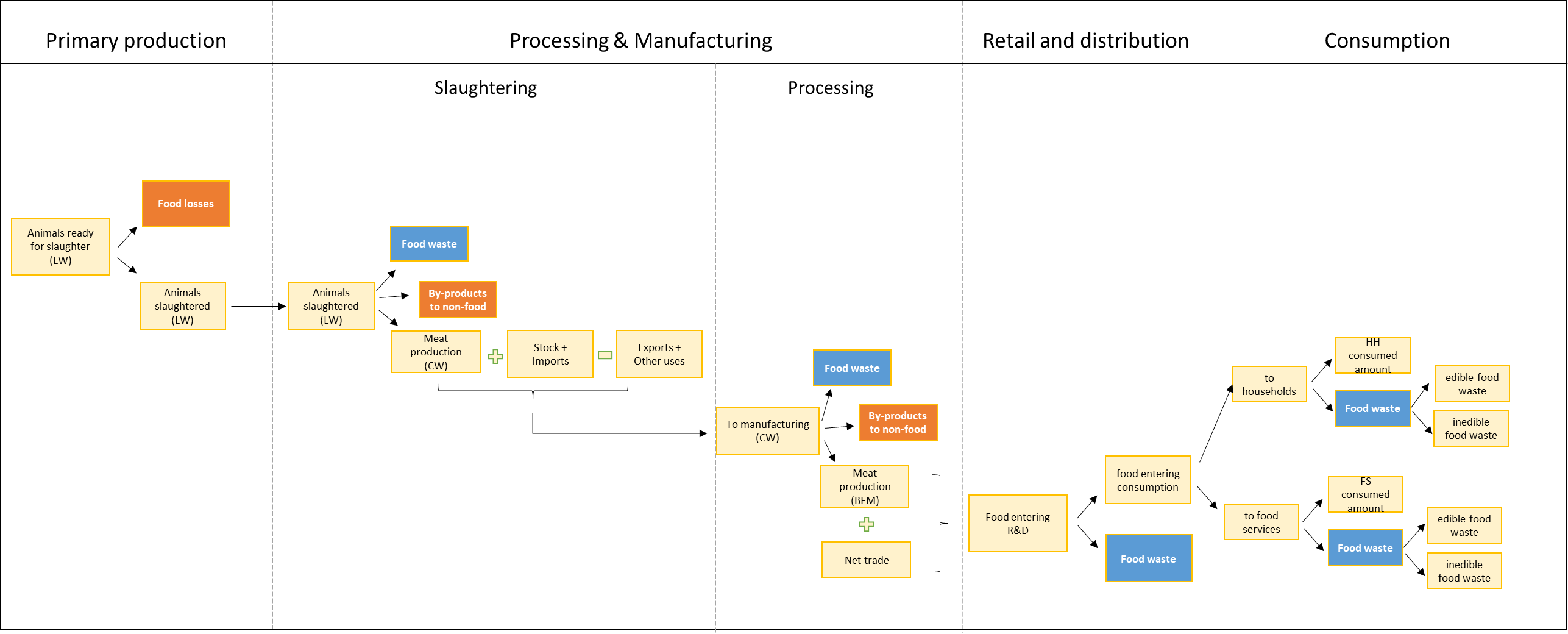


Figure 2. Conceptual scheme of the model developed (v1.0) for the meat food group. More details are provided in Section 1.1.2 (primary production) and Section 1.2.8 (processing and manufacturing). LW = live weight, CW = carcass weight, BFM = bone free meat.


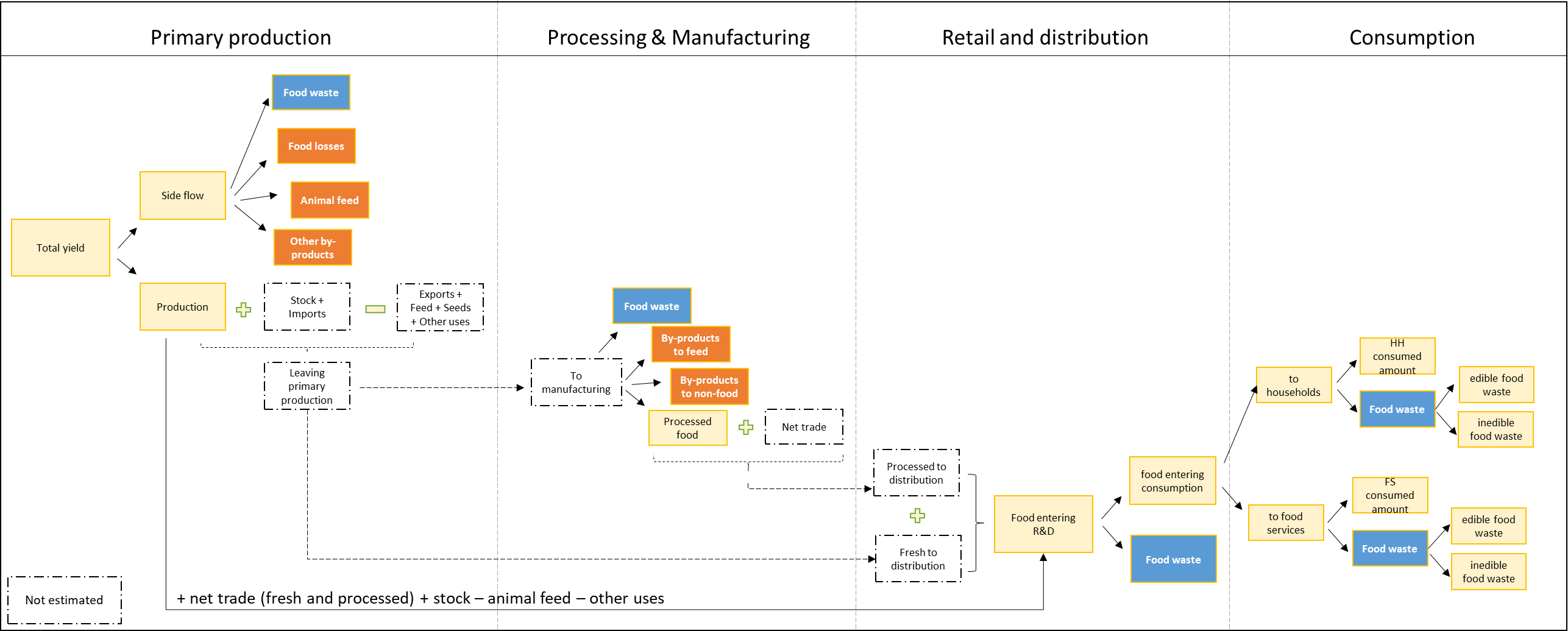


Figure 3. Conceptual scheme of the model developed (v1.0) for the fish food group. Trade data is taken from FAO CBS/FBS and refers to primary commodities and processed products expressed in live weight equivalent. The manufacturing stage is modelled separately than the other stages and the amount of fresh fish entering the manufacturing stage is not quantified. At primary production, retail and distribution, and consumption, items are expressed in live weight equivalent, while at processing and manufacturing items are expressed considering their real weight.

- 1. Primary production (PP)

The amount of crops and livestock products produced yearly in each MS is taken from FAOSTAT, specifically from the commodity balance sheets (CBS) for the years 2002-2013 and from the new food balance sheets (FBS) for the years 2014-2017^[[1]](#footnote-1)^. Additionally, live animals slaughtered in the EU are extracted from FAOSTAT livestock primary database. The crops and animal products included in each food group at this stage of the FSC are presented in **Table** 1. The detailed list of all the crops and animal products included at this stage, identified with their specific code in the source database, is provided in Annex 2, tables A2.1, A2.2, A2.3 and A2.4, for food items identified by FSC stage PP.

Table 1: List of crops and animal products included in each food group at primary production

| **Food group** | **Crops and animal products included at primary production (from FAO classification)** |
| --- | --- |
| Sugarbeet | Sugarbeet |
| Oilcrops | Olives; sunflower seed; rape and mustard seed; soybean; cotton seed |
| Fruit | Apples; bananas; citrus, other; dates; fruits, other; grapefruit; grapes; lemons, limes; oranges, mandarines; pineapple; plantains |
| Vegetables | Onions; tomatoes; vegetables, other; beans; peas; pulses, other |
| Potatoes | Potatoes; sweet potatoes |
| Cereals | Wheat; barley; rye; maize; oats; rice^[[2]](#footnote-2)^; other cereals |
| Dairy | Milk |
| Eggs | Eggs |
| Meat | Bovine; sheep; pig; poultry |
| Fish | Cephalopods; crustaceans; demersal fish; freshwater fish; marine fish; other molluscs; other pelagic fish |

- - 1. Plant based food groups: Sugar beet, Oilcrops, Fruit, Vegetables, Potatoes, Cereals, Oilcrops

A three-stepped approach was followed to calculate the food waste at primary production of plant-based food groups and the amounts of each entering the following stage of the supply chain (i.e. processing and distribution stages). These steps include: 1) the calculation of the amount of each crop produced for food purposes; 2) the calculation of food waste at primary production, and 3) the calculation of the amount of each crop leaving primary production (as illustrated in Figure 1). A detailed explanation of each step follows.

Step 1: calculation of the amount of each crop produced for food purposes

The CBS and the FBS provide information on the supply and uses of **crops** expressed in primary equivalent (i.e. the amount of primary commodity input that would be required to produce a given amount of derived product output (GSARS, 2017)). Table 2 provides an example of this information for the item: “wheat and products”. The domestic supply quantity (DSQ) is calculated as production plus imports plus stock variation minus exports (items highlighted in orange in Table 2**Table** 2). In this table, imports and exports quantities refer to trade flows of wheat and of wheat-derived products (e.g. flour) measured in primary equivalent (i.e. amounts of wheat necessary to produce the imported quantity of flour). The DSQ is then distributed amongst the following uses: feed, seeds, losses, processing, food supply quantity, other uses (items highlighted in green in Table 2). The information extracted from the CBS and FBS for this exercise is the following: production amount, stock variation, domestic supply quantity, feed, seeds and other uses. The last three items are then used to calculate the share of feed (S_f_), seeds (S_s_), and other uses (S_o_) of the domestic supply quantity, according to the following equations:

$S_{f}=\frac{Feed}{DSQ} [\%]$ [1]

$S_{s} =\frac{Seeds}{DSQ} \left[ \% \right]$ [2]

$S_{o} =\frac{Other uses}{DSQ} \left[ \% \right]$ [3]

Table 2: Example of commodity balance sheets (FAOSTAT), for the item "wheat and products", EU28, year 2013. All quantities are expressed in commodity primary equivalent.

| **Element** | **Value [Mt]** |
| --- | --- |
| Production | 144.4 |
| Import Quantity | 41.3 |
| Stock Variation | 2.3 |
| Export Quantity | 66.8 |
| Domestic supply quantity | 121.4 |
| Feed | 46.6 |
| Seeds | 5.3 |
| Losses | 3.2 |
| Processing | 4.9 |
| Food supply quantity (tonnes) | 53.4 |
| Other uses | 8.0 |

According to the definition of food waste, the calculation of food waste generated at primary production should only refer to the portion of harvested crops destined for human consumption. However, this information is not provided directly from the CBS/FBS. Hence, the amount of each crop harvested destined for food production (Pf) was calculated as follows:

$P_{f} = P -(P\times s_{f}+ P\times s_{s}+ P\times s_{o})$ [4]

Where P is the production from CBS/FBS, and s_f,_ s_s_ and s_o_ are calculated by means of equations 1, 2, and 3.

The underlying assumption is that the relative share of the considered uses (food, feed, seeds, and other uses) of the domestic supply quantity, which includes imported goods and excludes exported goods, as provided by the CBS/FBS, can be taken as a proxy of the relative share of uses of the crops produced in the region considered. In other words, the amounts that are used for feed, seeds, and other uses, according to the FBS/CBS include the imported quantity of each crop and exclude the exported quantity, the assumption taken here is that the relative share of each use, prior to trade, can be considered the same.

The elements “processing” and “losses” (Table 2) are not considered in this calculation. This is because under “processing” are reported the quantities of a crop processed for food purposes but entering a different commodity item group (e.g. “beverages, fermented” made of wheat). The element “losses” provides an estimation of waste and losses taking place between the level at which primary production is recorded and the household level (excluded) (FAO, 2001). As the aim of this calculation is to obtain the total quantity of a certain crop available at primary production to be used for food purposes, it is not necessary to subtract these two quantities.

Step 2: calculation of food waste at primary production

In this second step, food waste at primary production is calculated by combining the amount of each crop harvested to be used for food production (P_f_) with coefficients taken from the literature. Additionally, the following elements are derived:

- Food losses (defined in this work as crops left on fields and ploughed in)
- Surplus food used as animal feed (in addition to the amount used as animal feed as reported by FAOSTAT in the FBS/CBS)
- Other by-products (surplus food at primary production used for other uses)

As the production amounts reported in the FBS/CBS do not include waste and losses at primary production, the “total yield” (i.e. the total crop ready for harvest) is calculated from the produced amount (Pf), by using a coefficient (PP01) as illustrated in equation 5 and Figure 4. This coefficient defined “side flow coefficient” (from Hartikainen et al., (2018)) represents the share of the total crop ready for harvest that is either wasted, lost, transformed in animal feed or in other by-products. Then, each component (food waste, food losses, animal feed, other by-products) is calculated from the total yield, using coefficients derived from the literature, following equations 6 to 9.

The calculation of food waste, food losses, animal feed and by-products are illustrated in equations 5 to 9.

$Total yield= \frac{P_{f}}{(1-PP01)}$ [5]

$Food waste= \frac{P_{f}}{(1-PP01)} \times PP02$ [6]

$Animal feed= \frac{P_{f}}{(1-PP01)} \times PP03$ [7]

$Food losses= \frac{P_{f}}{(1-PP01)} \times PP04$ [8]

$Other by-products= \frac{P_{f}}{\left( 1-PP01 \right)} \times PP05$ [9]

Where:

PP01 = side flow coefficient, equal to PP02 + PP03 + PP04 + PP05

PP02 = food waste coefficient

PP03 = animal feed coefficient

PP04 = food loss coefficient

PP05 = other by-products coefficient

The coefficients used and their data sources are provided in Annex 1, table A1.1 for each food group.

The first two steps of this procedure are illustrated by Figure 4.


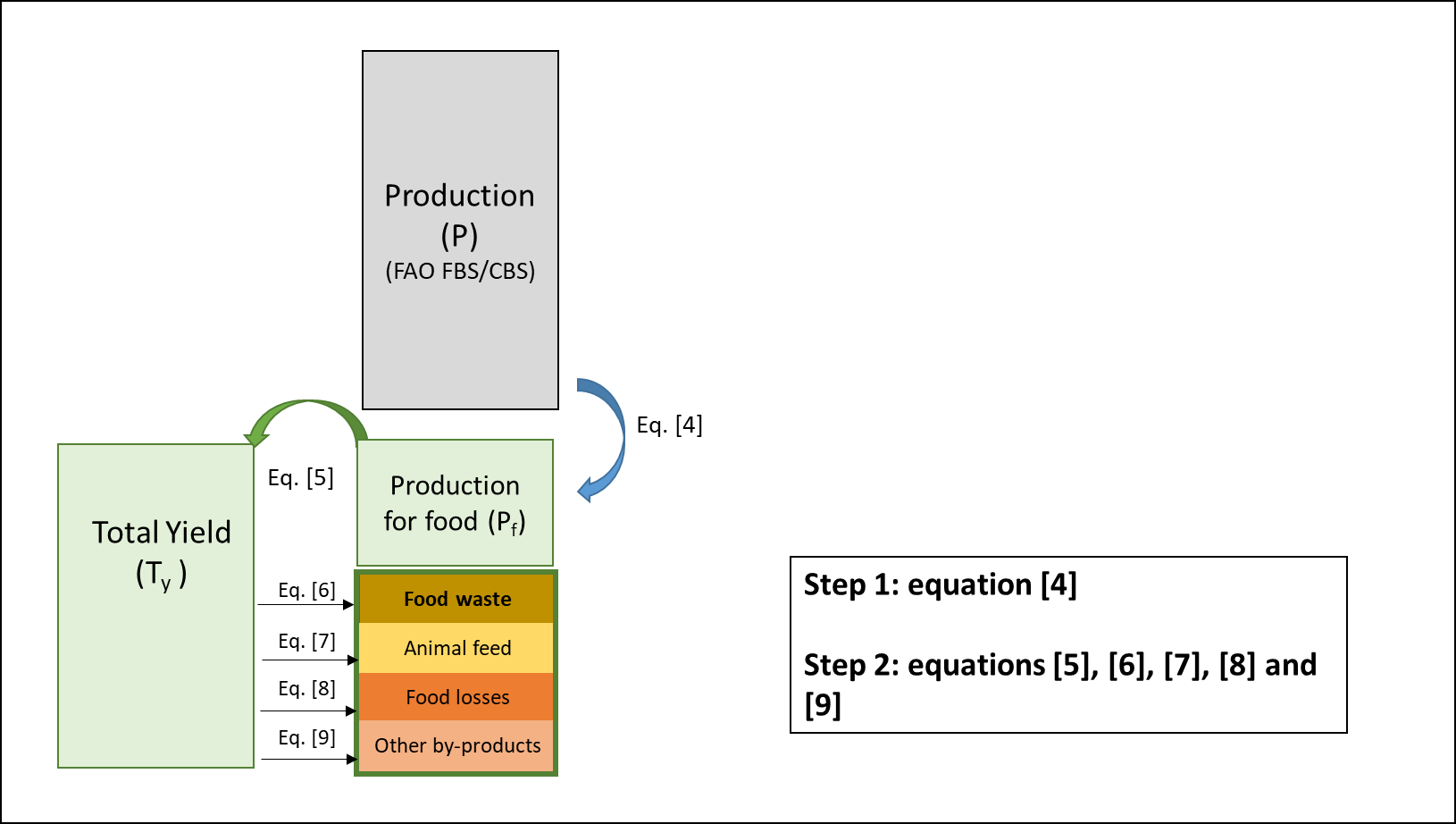


Figure 4: Visual representation of the calculation procedure to derive the amount of each crop produced for food, and the related food waste, animal feed, food losses and other by-products.

Step 3: calculation of the amount of each crop leaving primary production

Import and export quantities from the CBS/FBS present primary commodities together with processed products expressed in primary equivalent. Instead, the FAO trade database, reports imports and exports of primary and processed commodities separately (where the latter are expressed in their real weight and not in primary equivalent weight). An example of FAO trade statistics is provided in Table 3. It is possible to see, comparing Table 2 and Table 3, that imports and exports of wheat in the EU28 in 2013 were significantly lower than those of “wheat and products”, as it can be expected that most of the quantity of “wheat and products” imported refers in fact to processed products.

Table 3: Example of FAO trade statistics (FAOSTAT) for the item "wheat", EU28 (excluding intra EU trade), year 2013

| **Element** | **Value [Mt]** |
| --- | --- |
| Import Quantity | 3.9 |
| Export Quantity | 25.1 |

As the model developed distinguishes between food waste generated at primary production and food waste generated at processing and manufacturing, it is important to know how much of each crop is imported and exported in its unprocessed state (e.g. wheat), in order to calculate the amount of primary commodities leaving primary production to enter the processing and manufacturing stage and/or to enter directly the distribution stage to be distributed as fresh (this is the case only for certain food products such as fruit and vegetables) (as illustrated in Figure 1).

This calculation is performed as follows.

First, an “alternative domestic supply quantity” – DSQ*, is calculated following the same rationale of the domestic supply quantity from the CBS/FBS: as the sum of production quantity, stock variation, and net trade (imports minus exports). The reason for defining it “alternative” is that, in this case, a different source is used for trade data: instead of extracting trade data from the CBS/FBS, FAO trade statistics are used to extract trade data, as they provide this information for primary commodities only.

Equation 10 illustrates the calculation of the alternative domestic supply quantity (DSQ*).

${DSQ}^{*}=P + S + I - E$ [10]

Where P is the production (taken from CBS/FBS), S is the stock variation (taken from CBS/FBS), I and E are respectively imports and exports (taken from FAO trade).

Annex 2, Table A2.3 provides the list of codes for which data was extracted from FAO trade statistics. As the two databases use a different classification system, a mapping table is provided in Annex 2, Table A2.10, that links item codes in FAO CBS/FBS with item codes in FAO trade. This table is an input to the model.

The following equations (11 – 13) are then applied to calculate the portion of the DSQ* used for animal feed, seeds and other uses.

${Feed}^{*}= {DSQ}^{*}\times s_{f}$ [11]

${Seeds}^{*}= {DSQ}^{*}\times s_{s}$ [12]

${Other uses}^{*}= {DSQ}^{*}\times s_{o}$ [13]

Finally, the amount of crops leaving the primary production stage (Lpp), and therefore available for processing and fresh distribution, is calculated as follows.

$Lpp = {DSQ}^{*} -{Feed}^{*} - {Seeds}^{*}- {Other uses}^{*}$ [14]

The underlying assumption of this calculation is that the share of different uses is the same for the domestic supply calculated considering only raw commodities (DSQ*) and considering both raw and processed commodities (DSQ). This is a critical assumption, and might cause both underestimations or overestimations of the amounts leaving primary production, depending on how the actual distribution of uses of primary commodities is compared to the distribution of primary and processed commodities. For the same reasons presented under “Step 1”, and as the aim of equation 14 is to obtain the total quantity of a certain crop entering the processing stage for food purposes, the elements “processing” and “losses” from Table 2 are not considered.

The total animal feed generated at primary production is then calculated as the sum of two components: the animal feed calculated from equation 7 (as a share of the side flow) and animal feed calculated from equation 11 (based on CBS/FBS). Similarly, the item “other uses” calculated from equation 13 (based on CBS/FBS) is added to the quantity of “other by-products” calculated from equation 9 (as share of the side flow) to obtain the total amount of each crop at primary production destined to non-food uses.

For all plant based food groups, excluded oilcrops, the three steps presented above are applied directly to each crop included (presented in Table 1) to calculate:

- the amount of each crop used for food purposes,
- the related food waste, food losses, animal feed and other by-products generated at primary production, and
- the amount of each crop leaving the primary production stage and available for processing and fresh distribution.

Instead, the model was modified in the case of **oilcrops** to account for the significant non-food uses typical of this group of commodities. The modified version of the three steps presented above is reported here.

Step 1: calculation of the amount of oilcrops produced for food purposes

While for olive oil, the entire harvest is intended to be used for food purposes, the remaining oilcrops are used both for food production and biofuels production. The calculation on the amount of each crop used for food purposes, necessary to estimate the food waste and food losses in the food supply chain, is based on the share of vegetable oils used as biofuels in the EU in 2016 (OC1_i_), provided by Transport and Environment (2016) for the following vegetable oils: soybean oil, rapeseed oil, sunflower oil, palm oil, and other oil. Based on these shares, the amount of each oilcrop produced for food production is calculated by adapting equation 4 as follows:

$P_{f} =\sum_{i=1}^{5} \left[ P_{i} -\left( P_{i}\times s_{fi}+ P_{i}\times s_{si}+ P_{i}\times s_{oi} \right) \right]\times({OC1}_{i})$ [15]

Where: OC1_i_ is the share of vegetable oil used as food per type of oil (provided in Table 4), P_i_ is the produced amount of each oil (i), s_fi_, s_si_, s_oi_ are calculated following equations 1-3 for each oilcrop (i).

Figure 4 is also valid for oilcrops, if equation [4] is replaced by equation [15].

Table 4: Share of different oils used as biofuels in the EU in 2016 (Transport and Environment, 2017)

| **Oil type** | **Share for biofuel** | **Share for food (OC1)** |
| --- | --- | --- |
| Olive oil | 0% | 100% |
| Rapeseed oil | 60% | 40% |
| Sunflower oil | 4% | 96% |
| Soybean oil | 27% | 73% |
| Vegetable oil (generic) | 44% | 66% |

Step 2: calculation of food waste at primary production

Food waste, food losses, animal feed and other by-products are then calculated for the quantity of oilcrops used for food consumption (P_f_) using the coefficients reported in Annex 1, Table A1.1, by means of equations 5 to 9.

Step 3: calculation of the amount of each oilcrop entering the processing and distribution stages

The amount of each oilcrop leaving primary production is then calculated by adapting equation 14 as follows:

${Lpp}_{i} =({DSQ}_{i}^{*} -{Feed}_{i}^{*} - {Seeds}_{i}^{*}- {Other uses}_{i}^{*}) \times\left( {OC1}_{i} \right)$ [16]

Where ${DSQ}_{i}^{*}$, ${Feed}_{i}^{*}$, ${Seeds}_{i}^{*}$, and ${Other uses}_{i}^{*}$ are calculated for each oilcrop (i) by means of equations 10, 11, 12 and 13 respectively.

The total amount of oilcrops leaving primary production is then calculated as presented in equation 17.

$Lpp =\sum_{i=1}^{5} {Lpp}_{i}$ [17]

- - 1. Animal based food groups: Dairy, Eggs, Meat, and Fish

As for **animal products**, the modelling of milk and eggs follows the same approach presented for crops. The only differences are that in the case of milk the element “seeds” is equal to zero, while for eggs the element “feed” is equal to zero, whereas the element “seeds” accounts for eggs used for hatching. Instead, the modelling of fish and meat at primary production presents substantial differences to the other food groups. This is presented in detail below.

Meat

As specified in the main text, in this work primary production of the meat supply chain includes the activities taking place up to slaughtering, the latter being excluded as considered part of the processing stage. According to the EU legislation (European Parliament and Council, 2002) live animals are not considered to be food, therefore at this stage no food waste is produced. However, there are losses caused by mortality of the animals ready for slaughter, both during transport to slaughterhouse and rejects at slaughterhouse, which are here quantified and classified under food losses.

Food losses at primary production are calculated by applying food loss coefficients to the live weight of livestock slaughtered in each country (Pf). This is calculated from the number of animals slaughtered (Hi), as extracted from FAOSTAT livestock primary database (see Annex 2, Table 2.4), and the average weight of each species at slaughter (PP06), extracted for each country from the GLEAMi website^[[3]](#footnote-3)^, as illustrated by equation 18.

P_f_ = $\sum_{i} H_{i}\times{PP06}_{i}$ [18]

Where:

H_i_ are the number of heads of each species (i) slaughtered in the country considered

PP06_i_ is the average weight at slaughter of species (i) in the country considered

The full list of average weights at slaughter considered is provided in Annex 1, Table A1.1.

The calculation of food losses is done following equation [7]; however, for this food group the side flow coefficient and the food losses coefficient are the same (i.e. the full side flow is considered a food loss, PP01 = PP04), as illustrated in Figure 3. The coefficients used are provided in Annex 1, Table A1.1.

The amount of meat leaving primary production corresponds to the total live weight of livestock slaughtered in each country (Pf).

Fish

The CBS and the FBS provide information on the supply and uses of fish expressed in live weight equivalent. The following information is extracted from the FBS/CBS: production, imports, exports, animal feed and other uses. The same procedure presented for plant-based food groups (**step 1**) is then applied to calculate the amount of fish produced for food purposes (Pf), by means of equation 4.


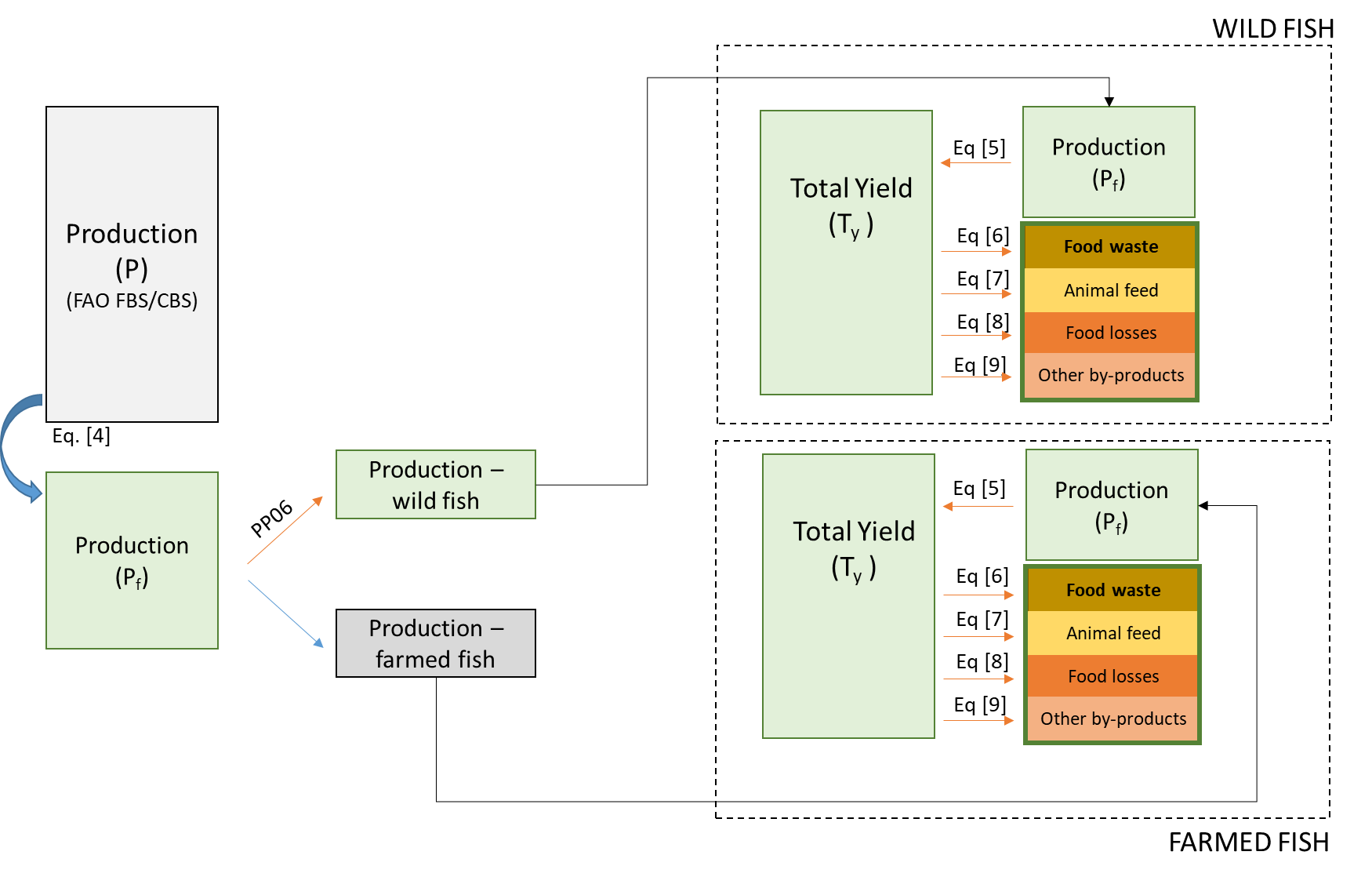


Figure 5: Visual representation of the calculation procedure to derive the amount of fish produced for food, and the related food waste, animal feed, food losses and other by-products

Then, as illustrated by Figure 5, a distinction is made between farmed fish and wild caught fish. This is because food waste coefficients at primary production from the literature study consulted are provided separately for wild fish and farmed fish. As such information is not provided in FAOSTAT, the distinction is made considering the share of wild and farmed fish provided by EUMOFA, (2018) for fish fished in the EU by species type. The table with the coefficients used is provided in Annex 1, Table A1.1, (coefficients PP06 for fish).

Food waste and by-products (in this case only animal feed) generated at primary production (i.e. fishing of wild and farmed fish) are then calculated by applying coefficients taken from the literature to the Pf, as illustrated in equations 5, 6, 7 and 8, following **step 2** of the procedure presented for plant-based food groups. The coefficients used are provided in Annex 1, Table A1.1. It is possible to see that not all coefficients are provided (PP01 and PP02 only for wild fish, and PP01 and PP03 only for farmed fish), as according to the source consulted (ADEME, 2016), the entire side flow generated from wild fish is wasted (by-catches thrown back in sea), while the entire side flow generated from farmed fish is used as animal feed. Therefore, in the model, food waste at primary production for this food group is only related to wild fish. It should be noted that the study used to estimate waste of wild fish (i.e. ADEME, 2016) is based on data collected in 2013. Between 2015 and 2019 European vessels were required to gradually eliminate the practice of discarding back to sea unwanted catches, through the introduction of the landing obligation introduced by the new Common Fisheries Policy (European Parliament and of the Council, 2013). Therefore, this coefficient is expected to be overestimating food waste generation for the years following 2015.

The calculation of the amount of fish entering the processing and distribution stages (step 3) is significantly different to what was presented for the other food groups (as reported in Figure 3). The reason for this is that, unlike the other food groups, it is not possible to distinguish between trade of fresh and processed fish products, as part of the processing already takes place at sea (gutting, freezing) and trade statistics report together raw and processed fish products. For this reason, it is not possible to quantify the amount of fish leaving the primary production stage and entering the processing stage, instead, based on FAOSTAT data, it is only possible to quantify directly the amount of fish entering the distribution stage, expressed in live weight equivalent.

The amount of fish entering the distribution stage is therefore calculated directly from the CBS/FBS as:

*Fish entering distribution = P + I + S – E – O – A* [19]

Where

P = production

I = import

S = stock variation

E = export

O = other uses

A = animal feed

Although it is not possible to quantify the amount of fresh fish entering the processing and manufacturing stage, an estimation of food waste generated at P&M is performed, as presented in section 1.2.9.

- 1. Processing and manufacturing (P&M)

Of the stages considered, the processing and manufacturing stage is the most complex to model, due to the large number of transformations that take place to derive processed food products from crops and animal products that leave the primary production stage. For this reason, this is also the stage where the modelling approach varies substantially across the different food groups. A detailed description of the model is provided in the sub-sections below per food group.

An assumption common to several food groups is that, due to lack of data, inefficiencies of the processing stage causing food waste and by-products are often not considered (this is not the case for potatoes and cereals, where some information on the efficiency of transformation processes could be retrieved). When this is the case, and inefficiencies are not accounted for, food waste and by-products are estimated considering the inedible component of different commodities. This implies that the food waste and by-products generated at processing are most likely underestimated.

Furthermore, it is expected that at this stage of the FSC, the use of side streams from food processing activities for several purposes (e.g. animal feed production, production of biomaterials, other non-food uses) is a common practice, as a way to maximise profit and reduce costs related to waste treatment operations. In this case, following the definition adopted in this work, residues would be classified as by-products. However, it is quite challenging to estimate the relative share of by-products and of waste generated by the different food manufacturing sectors, due to a lack of statistical data providing this information. For this reason, the amount of food waste estimated at this stage is affected by the assumptions made on what share of the residues generated will become a by-product, and might consequently be both underestimated or overestimated.

The main inputs of the modelling approach adopted at this stage are statistical data on production and trade of processed food products taken from a range of databases from Eurostat, FAOSTAT and EUMOFA^[[4]](#footnote-4)^ websites (i.e. FBS, CBS, Prodcom, APRO, FAO trade, COMEXT, EUMOFA) and coefficients taken from the technical literature. Annex 2 provides for each source of data the full list of items extracted at this stage (identified with FSC stage P&M), in Tables A2.1, A2.2, A2.3, A2.5, A2.6, A2.7, A2.8 and A2.9. Additionally, the full list of coefficients used in the calculations at P&M is provided in Annex 1, Table A1.2.

In some cases, in order to deal with data gaps in the Prodcom database for trade of processed products, an alternative option from where to extract the data is provided. In other words, if no data is available from the first data source (Prodcom), a second data source can be used in replacement (FAO trade). To this end, a mapping between the item codes of the two different data sources is provided as input to the model and can be found in Annex 2 Table A2.11.

- - 1. Sugar beet

The entire flow of sugar beet leaving the primary production stage, calculated from equation 14 (section 1.1.1) is assumed to enter to processing and manufacturing stage (i.e. no sugar beet entering the distribution stage as such). Figure 6 illustrates the modelling approach adopted for this commodity. From the amount of sugar beet entering the processing stage, it is assumed that when processed into sugar 78% of the initial weight is water leaving the system (i.e. due to evaporation) – (coefficient SU2 in Figure 6).

Waste and by-products are then calculated by means of mass balance, as the difference between the quantity of sugar beet entering the processing stage, the water evaporating and the amount of refined sugar produced. The latter is taken from the CBS for all years until 2013, where it is provided under the classification “Sugar, refined equivalent”. As the FBS do not provide such information, for all years after 2014, the value of production of “Sugar, raw equivalent” is extracted from the FBS, and a coefficient of 92% is used to calculate the equivalent quantity of refined sugar (coefficient taken from FAO, (2003)).

Waste and by-products are then differentiated by means of coefficients. All coefficients are provided in Annex 1, Table A1.2.


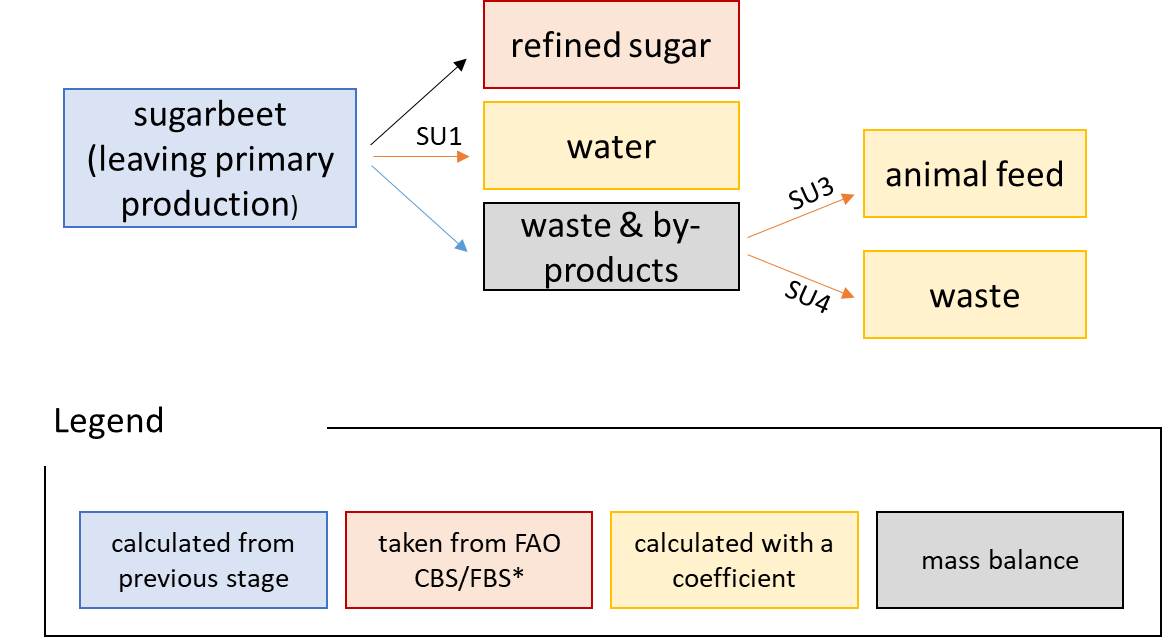


Figure 6: Modelling of the processing stage of sugar beet into refined sugar. * the amount of refined sugar is taken from CBS until 2013. As the FBS does not provide this information, for the years after 2013 refined sugar is estimated by multiplying the quantity of raw sugar equivalent by a coefficient SU2 (SU2 = 92%).

Trade data of processed sugar is taken from FAO trade statistics considering both “Refined sugar” and “Sugar raw centrifugal” (this is an intermediate product that is further processed to become refined sugar). After converting the latter into its refined equivalent quantity (using a coefficient of 92%), total imports and exports are calculated by adding together refined sugar and raw sugar converted into refined equivalent.

This information is then used to calculate the amount of refined sugar entering the distribution phase (E_d_), identified as “Processed to distribution” in Figure 1, according to the following equation.

$Ed = P + S + I - E - O$ [20]

Where:

P = production of refined sugar (taken from CBS/FBS)

S = stock variation of refined sugar (taken from CBS/FBS)

I = total imports of refined sugar and raw sugar converted in refined equivalent (taken from FAO trade)

E = total exports of refined sugar and raw sugar converted in refined equivalent (taken from FAO trade)

O = other uses (taken from CBS/FBS)

- - 1. Oilcrops

The following products are modelled at this stage: preserved olives, olive oil, rapeseed and mustardseed oil, soybean oil, sunflowerseed oil, cottonseed oil. The amount of oilcrops leaving primary production to enter the processing stage for food production is calculated as presented in section 1.1.1 (equations 16 and 17). Similarly to sugar beet, no oilcrops are assumed to enter the distribution phase in their unprocessed state. The modelling of waste and by-products at the processing stage varies according to oil type.

For rapeseed and mustard seed oil, soybean oil, sunflower seed oil, and cotton seed oil, the amount of each vegeable oil produced (P_i_) is taken from the FAO CBS/FBS and multiplied by a specific coefficient (coefficients OC1_i_ in Table 4) to derive the amount produced for food purposes (P_f,i_).

$P_{f,i} = P_{i} (CBS/FBS) \times{OC1}_{i}$ [21]

Then, as illustrated in Figure 7, the cake generated at processing and manufacturing is calculated by means of mass balance by subtracting from the amount of each oilcrop entering the processing stage (calculated following equation 16) the amount of vegetable oil produced for food purposes. As can be seen from the coefficients provided in Annex 1, currently the model assumes that all the cake generated is used as animal feed (in other words OC3 is equal to 100% and OC2 is equal to 0%). According to the model, the processing of vegetable oils for the above mentioned oilcrops does not generate food waste.


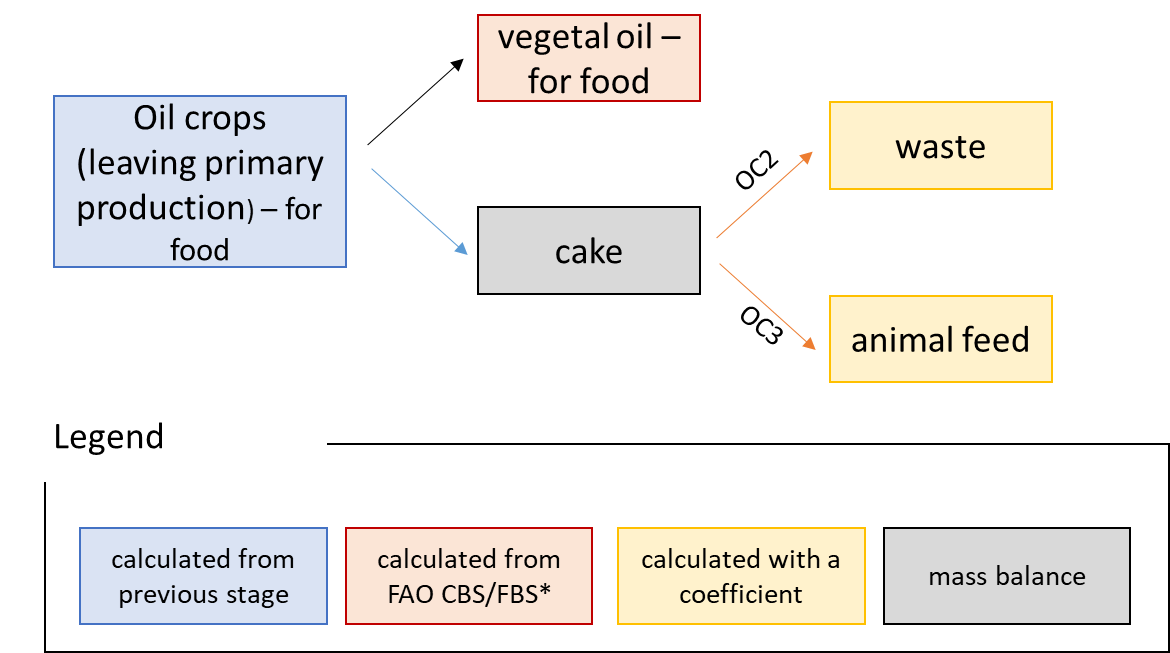


Figure 7: Modelling of the processing stage of oil crops (rapeseed and mustard seed, soybean, sunflower seed, and cotton seed) into vegetable oils. * Calculated as: amount reported in CBS/FBS x OC1 (Table 4)

The procedure for olives is illustrated by Figure 8. The amount of preserved olives produced is extracted from Prodcom. Then, from the total olives leaving primary production (calculated following equation 16 in section 1.1.1) the preserved olives are subtracted, providing the amount of olives transformed into olive oil. Coefficients derived from (Consejería de Agricultura y Pesca, 2015) are used to estimate from this quantity:

- the olive oil produced (including both olive oil and olive pomace oil),
- the amount of pomace and olive pits resulting from the extraction of olive oil and a subsequent extraction of olive pomace oil and
- the wastewater generated.

Waste and by-products are then calculated from the quantity of pomace and olive pits, following (Consejería de Agricultura y Pesca, 2015). All coefficients used are provided in Annex 1, Table A1.2.


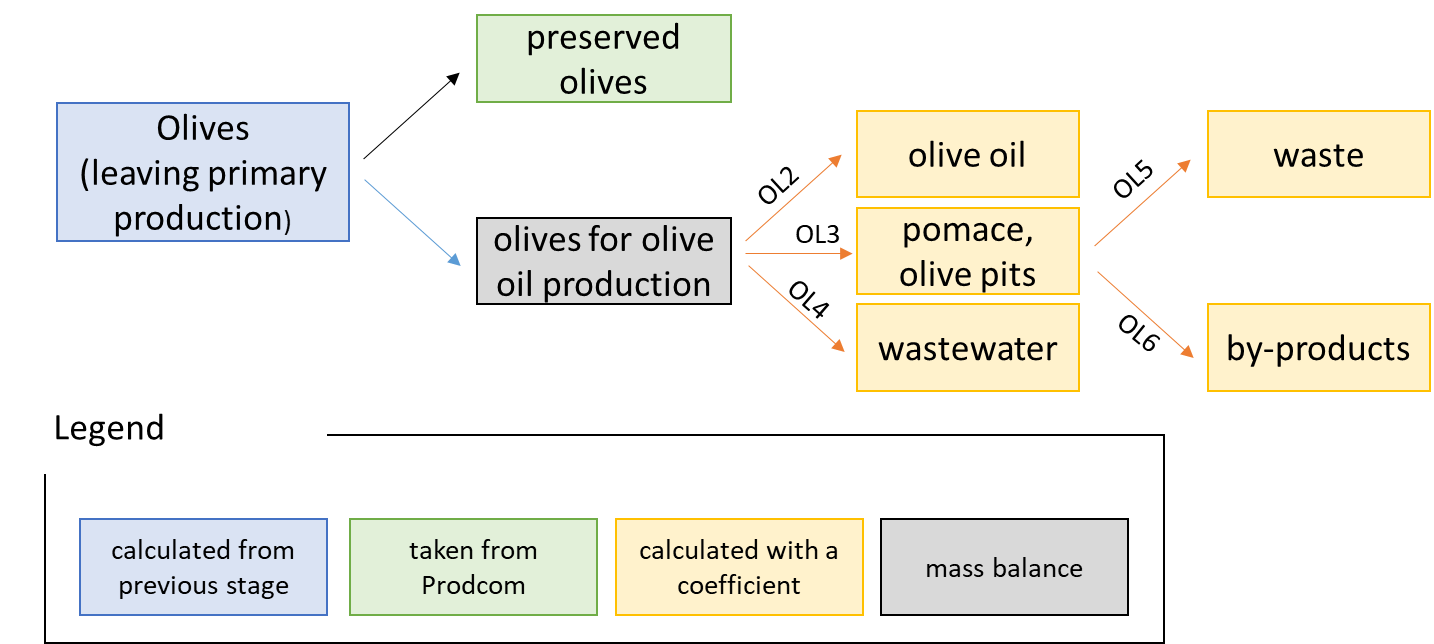


Figure 8: Modelling of the processing stage of olives into processed olives and olive oil. *Calculated as: amount reported in CBS/FBS x OC1 (Table 4)

Finally, the amount of processed olives, and vegetable oils (including olive oil) entering the distribution phase (“Processed to distribution” in Figure 1) is calculated by adding to the amount produced for food the net trade of each product. Net trade is calculated as imports minus export taken from FAO trade statistics. At this stage, in addition to the vegetable oils considered above, also the net trade of palm oil is taken into account. This is because, according to FAOSTAT, palm oil is not produced in EU MSs but is imported from extra EU countries.

For all vegetable oils (thus including palm oil), only the share of the net trade used for food is considered, to this end the coefficients OC1_i_ from Table 4 are applied.

- - 1. Potatoes

The amount of potatoes leaving primary production is calculated by means of equation 14.

Data on processed potato products at Member State level is affected by several gaps due to confidentiality issues. This affects the availability of processed potato products in the Prodcom database for several countries. Furthermore, there is no statistical information on the amount of fresh potatoes entering the manufacturing stage, nor on the amount of fresh potatoes distributed as such. For this reason, an alternative approach to the one adopted at EU level in Caldeira et al., (2019b) was adopted in this update of the model.

Based on data provided by EUPPA, (2020) on the amount of potatoes used by the manufacturing sector to derive processed potato products and the mass of final products produced in the main EU producing countries (i.e. Belgium, the Netherlands, France, United Kingdom and Germany), and on the amount of potatoes leaving primary production in each MS, it was possible to estimate three coefficients used in the model:

- the percentage of the amount leaving primary production that enters the P&M stage for the 5 main producing countries (equal to 61%)
- the percentage of the amount leaving primary production that enters the P&M stage in the remaining countries (equal to 18%)
- the average ratio between the final potato products and the raw potatoes entering the P&M stage (equal to 53%)

Based on these coefficients, it is possible to calculate for each country: the amount of potatoes entering the P&M stage (“to manufacturing” in Figure 1), the amount of processed potato products leaving the manufacturing stage (“processed food” in Figure 1), and the amount of fresh potatoes entering the distribution phase (“fresh to distribution” in Figure 1). The latter element is calculated by performing a mass balance, subtracting from the potatoes available for processing and distribution the ones used by the manufacturing industry, as illustrated in Figure 9.

This approach was selected even though it is a coarse approximation of what happens in reality (as even between the five main producing countries the share of potatoes directed to manufacturing is quite variable), based on considerations on data availability and in order to protect data confidentiality.

Waste and by-products generated at the processing stage are then derived from the amount of potatoes processed, as illustrated in Figure 9. All the coefficients used to model the processing of potatoes, reported in Annex 1, Table A1.2, were derived from the information provided by EUPPA, (2020).


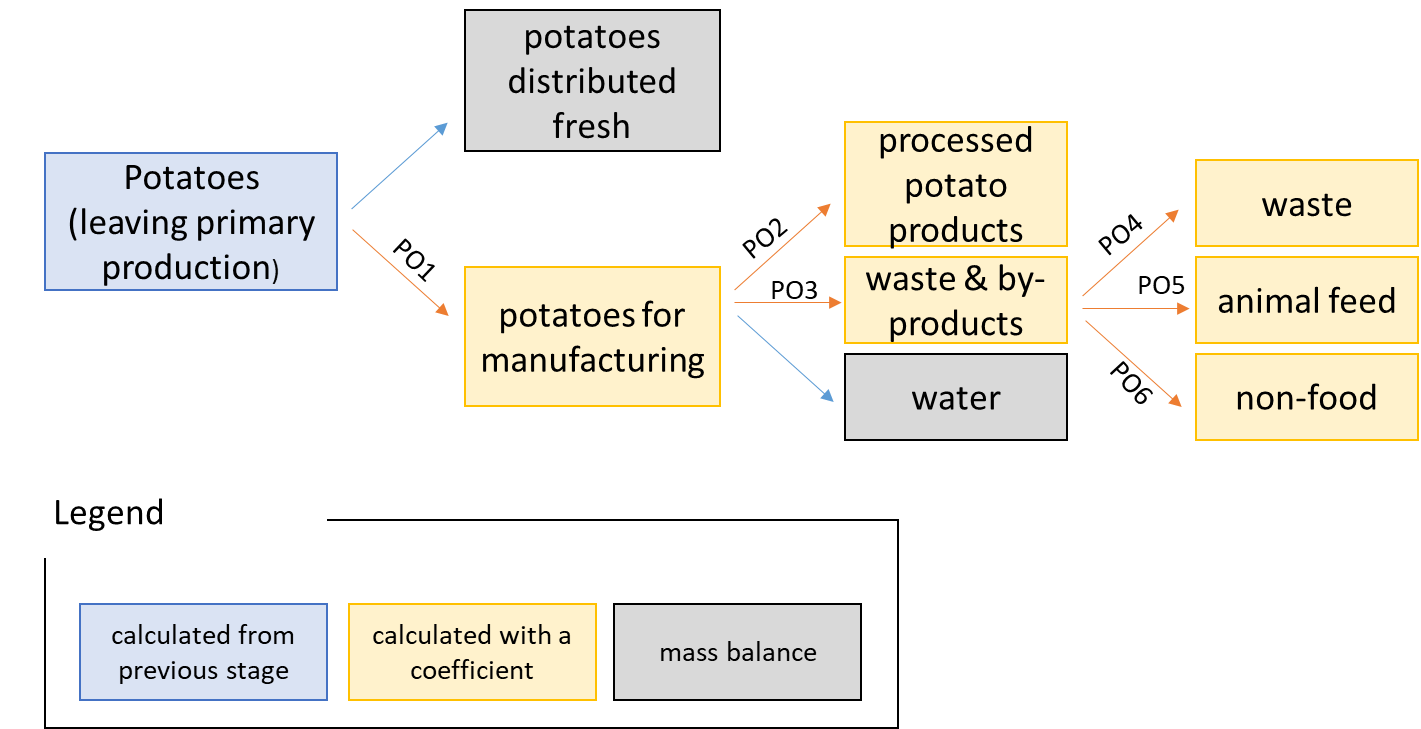


Figure 9: Modelling of the processing stage of potatoes.

The net trade of processed potato products was then derived from Prodcom (see Annex 2, Table 2.5). Finally, the total flow of potatoes and products entering the distribution phase was calculated by adding together the potatoes entering the distribution stage as fresh, the processed potato products produced and the net trade of processed potato products (as illustrated in Figure 1).

- - 1. Fruit & Vegetables

Food groups fruit and vegetables at P&M were modelled in the same way and are therefore described together in this section.

Of the amount of fruit and vegetables leaving primary production, calculated by means of equation 14, a part will directly be distributed fresh, and the rest will be processed into wine and other fruit-based products (e.g. juice, jams) or vegetable-based products (e.g. tomato sauce), as reported in Figure 1. The modelling of fruit and vegetables at this stage is illustrated respectively in Figure 10 and Figure 11.


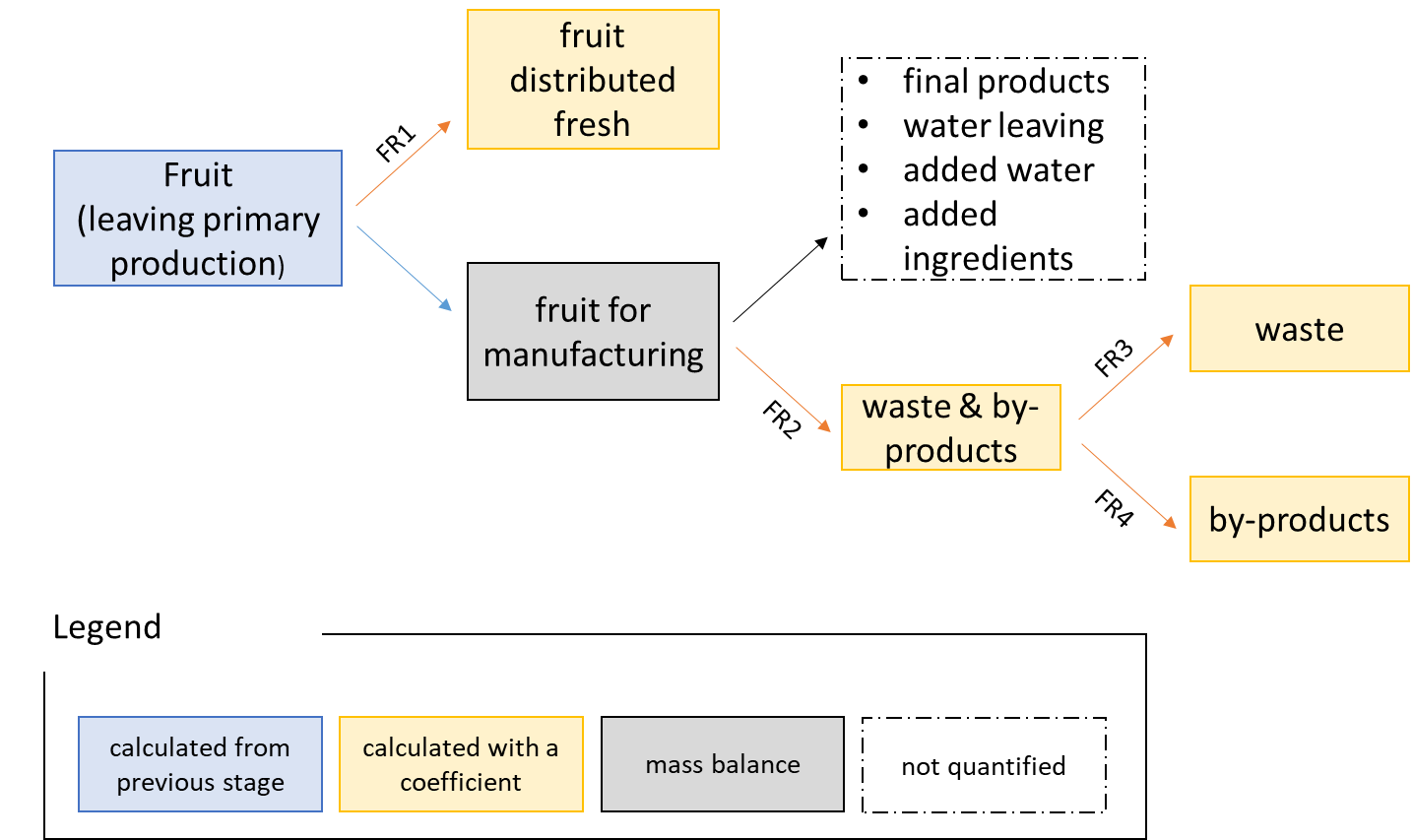


Figure 10: Modelling of the processing and manufacturing stage of fruit


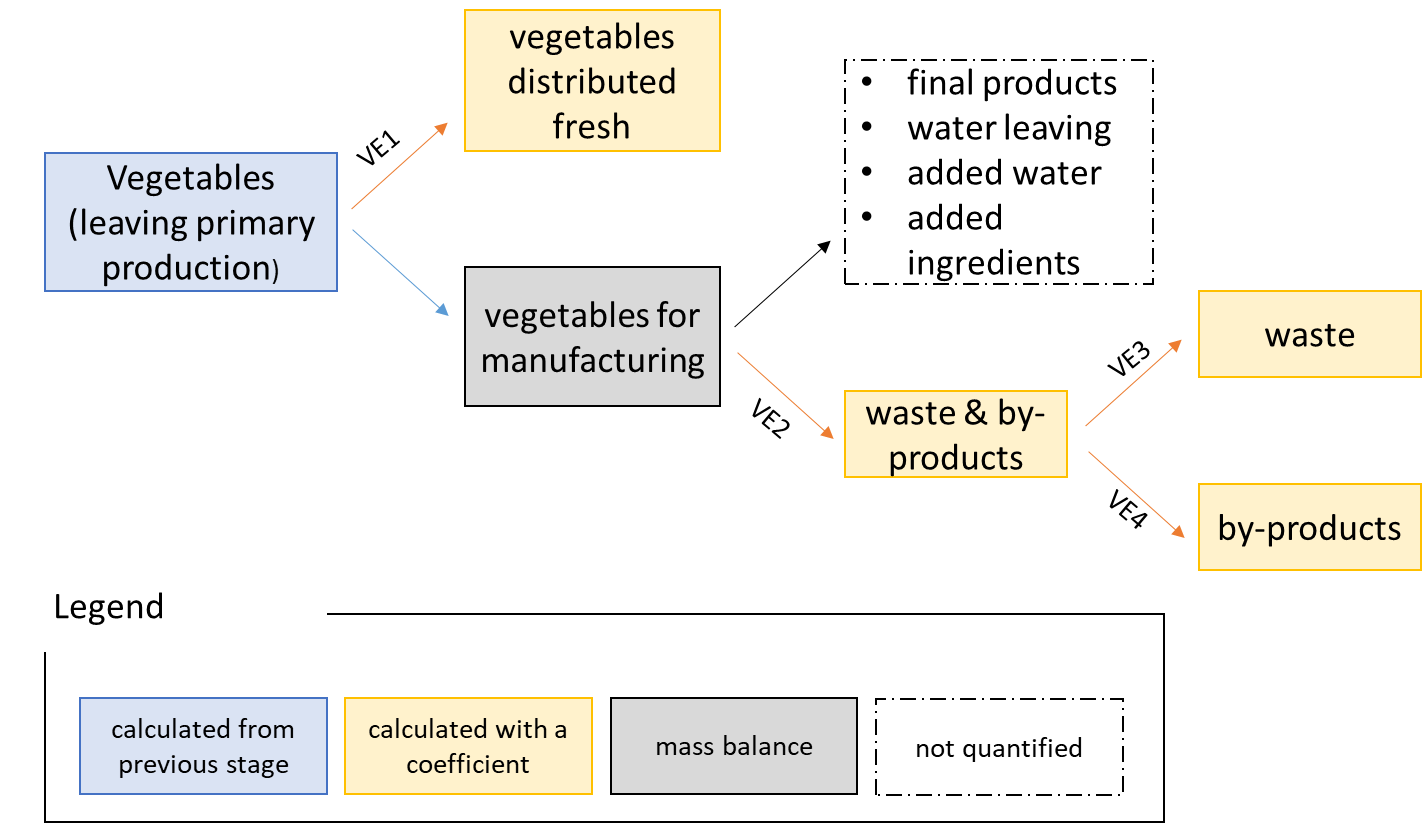


Figure 11: Modelling of the processing and manufacturing stage of vegetables

As there is no official statistics on the amount of fruit/vegetables distributed fresh, nor on the amount fruit/vegetables entering the manufacturing stage, the share of each flow in the different MSs needed to be estimated.

Information provided in Freshfel, (2019) on the amount of fruit/vegetables consumed fresh in the MSs in 2017 was used to perform this estimation. From the quantities of fresh fruit/vegetables consumed per capita, the total amount of fresh fruit/vegetables distributed was calculated for each country. Then this quantity was divided by the amount of fruit/vegetables leaving primary production in 2017, calculated by means of equation 14, to calculate two coefficients (FR1 in Figure 10, and VE1 in Figure 11) for each country that represents the share of fruit/vegetables leaving primary production to be distributed fresh. As this information is only available for the year 2017 (fresh fruit/vegetables consumed from (Freshfel, 2019)), these coefficients are considered static and are applied to all years. In one case (fresh fruit to distribution coefficient calculated for Lithuania), this process yielded a coefficient larger than 1 and the coefficient was assumed to be equal to 1 (meaning that no fruit is considered to be processed in that country). This is reasonable in countries that are not producing significant amounts of fruit at primary production, that are more likely to import directly the processed fruit products (e.g. wine, or juice) rather than importing the raw materials to produce domestically processed fruit products). The coefficient FR1 calculated for each country are provided in Annex 1, Table A1.2.

The amount of fruit/vegetables used at P&M is then calculated by performing a mass balance as the amount leaving primary production minus the amount distributed fresh.

To estimate the food waste generated when producing processed fruit/vegetables products, the amount of fruit/vegetables used at manufacturing is multiplied by coefficients (FR2 and VE2), equal to the average inedible component of fruit/vegetables according to De Laurentiis et al., (2018). The waste and by-products derived from this quantity are obtained by means of coefficients taken from (Kemna et al., 2017). All coefficients used are provided in Annex 1, Table A1.2.

As can be seen from Figure 10 and Figure 11, the transformation of fresh fruit/vegetables into the final processed products is not modelled due to: i) the complexity of the different transformations and ii)the lack of data on the amount of each type of fruit/vegetables available for processing (or available for fresh distribution). Instead, the processed fruit/vegetable products produced and traded by each country are extracted from Prodcom with the exception of wine which is taken from FAOSTAT CBS. The imports and exports of processed fruit/vegetable products are taken from FAOSTAT trade statistics in case of data gaps in the Prodcom database (wine is instead taken from FAOSTAT trade by default due to several data gaps found in Prodcom). Then the processed fruit/vegetable products entering the distribution phase are calculated as the sum of production and net trade.

The total amount of fruit/vegetables and fruit/vegetable products entering the distribution phase are then calculated by adding together the fresh fruit/vegetables and processed fruit/vegetable products entering this stage, as illustrated in Figure 1.

- - 1. Cereals

The processing stage of cereals is modelled considering the processing of the following crops: common wheat, durum wheat, barley, maize and rice. The amount of wheat (common plus durum wheat), barley, maize and rice available for processing is calculated as explained in Section 1.1.1 (equation 14). As in FAOSTAT CBS/FBS the distinction between common wheat and durum wheat is not provided, the amount of durum wheat produced is extracted from Eurostat APRO database, and the amount traded from COMEXT. Finally, the amount of durum wheat used for animal feed production and for seeds are estimated as, respectively, 7% and 5% of the amount produced (coefficient derived from the cereal balance sheets for 2015 taken from Eurostat database). Based on this, equation 14 is applied to calculate the amount of durum wheat available for processing. The amount of common wheat available for processing is obtained by subtracting the latter from the flow of wheat available for processing.

The modelling of the processing of cereals into cereal based products is mostly based on the model of the cereal supply chain developed by Courtonne et al., (2015b), from which coefficients were derived and applied to the amount of each cereal available for processing. This source provided information on the different uses of cereal crops in France, and on the efficiency of the processes of converting crops into derived products. The distinction between waste and by-products generated was instead based on ADEME (2016). The modelling approach adopted is presented below differentiating by crop type. All coefficients used are provided in Table A1.2 of Annex 1.

Figure 12 illustrates the modelling of the transformation of common wheat into starch and flour, and the transformation of flour into its derived products. Coefficients are used to derive the amount of common wheat used by the starch and glucose industry and the amount processed into flour, then additional coefficients are used to derive the amount of waste and by-products generated in these transformations. Then the flour produced is added to the net trade of flour, and coefficients are used to distinguish between the different uses of flour and the waste and by-products generated.


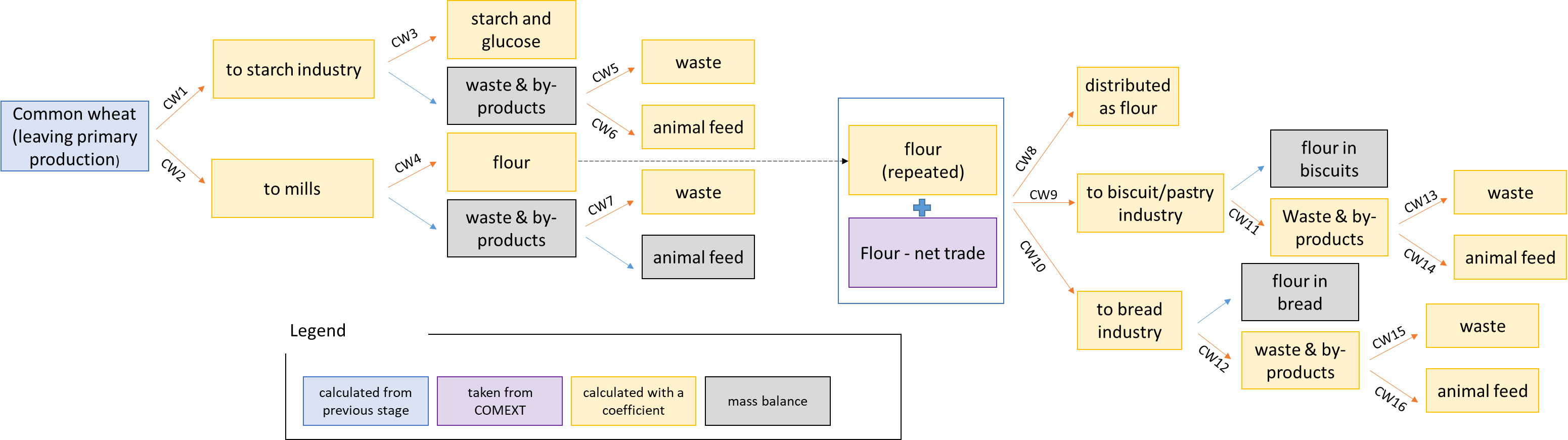


Figure 12: Modelling of processing and manufacturing stage of common wheat.

Figure 13 illustrates the modelling of durum wheat into semolina and then into pasta and couscous. Durum wheat entering the P&M stage is calculated as the sum of the produced amount taken from APRO database (see Annex 2, Table A2.6, plus net trade (from COMEXT, see Annex 2, Table 2.8), minus 5% of production (seeds), minus 7% of production (feed). Coefficients are used to derive the amount of semolina produced, and the waste and by-products are derived by means of mass balance. Then, the semolina produced is added to the net trade of semolina, and the amount of derived pasta and couscous are estimated, together with the waste and by-products generated in the process. The by-products generated are assumed to be used as animal feed, according to ADEME (2016).

Figure 14 illustrates the modelling of maize wheat into starch and glucose. First, the amount of maize used for the production of starch and glucose is derived by means of a coefficient. The remaining amount, calculated with a mass balance, is assumed to be used by the cornmill and canned corn industry. These processes are not modelled further. Coefficients are then used to calculate the final amount of starch and glucose produced, together with the waste and by-products generated in the process. The by-products generated are assumed to be used as animal feed, according to ADEME (2016).


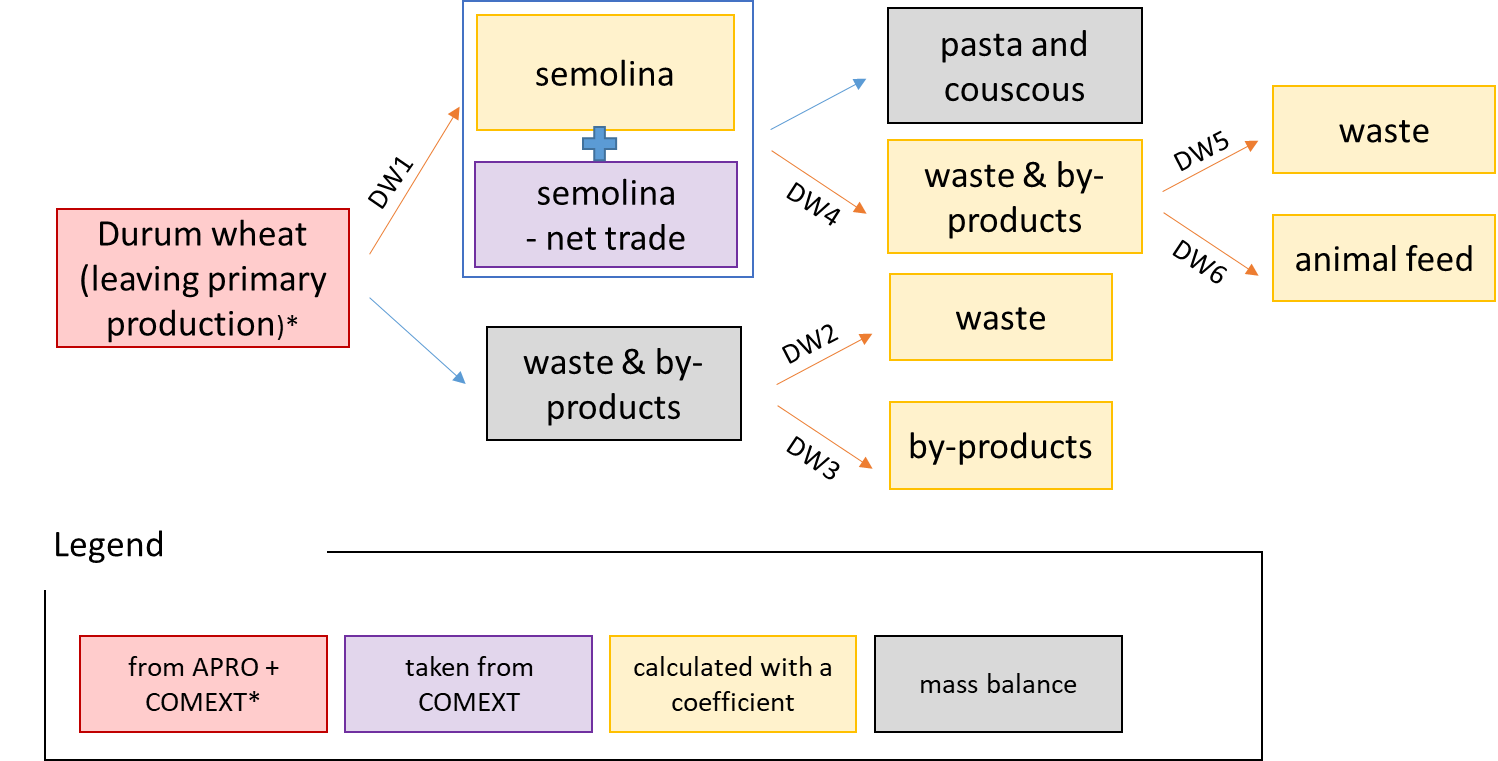


Figure 13: Modelling of durum wheat into pasta and couscous. *calculated from APRO, plus net trade (from COMEXT), minus 7% of production (feed), minus 5% of production (seeds) (respectively DW7 and DW8 in Annex 2, Table A1.2).


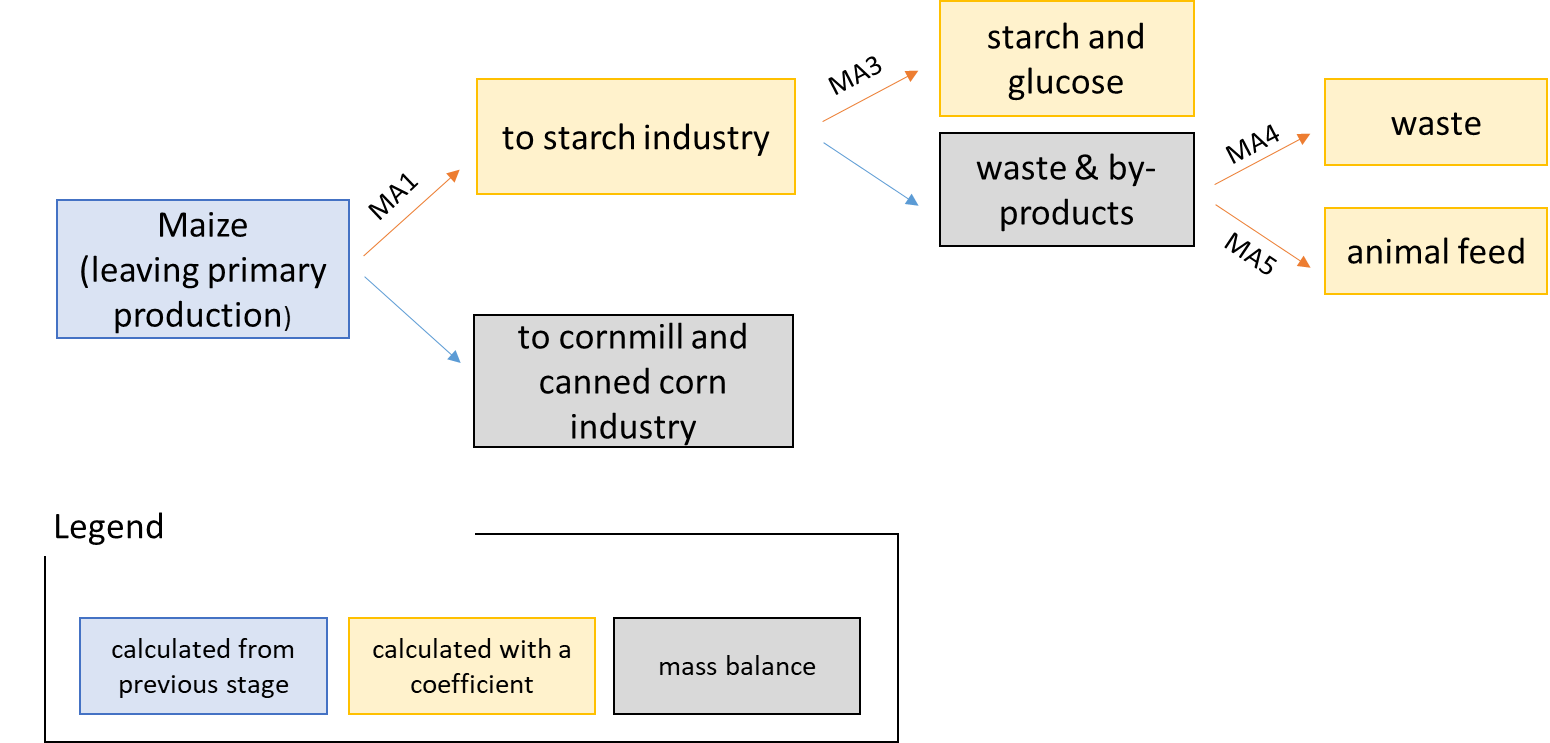


Figure 14: Modelling of maize into starch and glucose, and cornmill and canned corn

The modelling of rice is presented in Figure 15, coefficients are used to derive the amount milled rice produced and to derive the waste and by-products generated in this process. All by-products are assumed to be used as animal feed.

The modelling of barley is illustrated by Figure 16. In a first transformation, barely is processed into malt. Here, coefficients are used to derive the amount of malt produced, and the amount of waste and by-products, while the water leaving this system is calculated by means of mass balance. The process of transforming malt into beer is not modelled, due to missing data on added water and added ingredients. Therefore, from the quantity of beer produced (derived from Prodcom), using specific coefficients derived from (Roth et al., 2019), the amount of brewer’s spent grain generated is estimated (assumed to be used as animal feed) and the amount of waste is obtained.


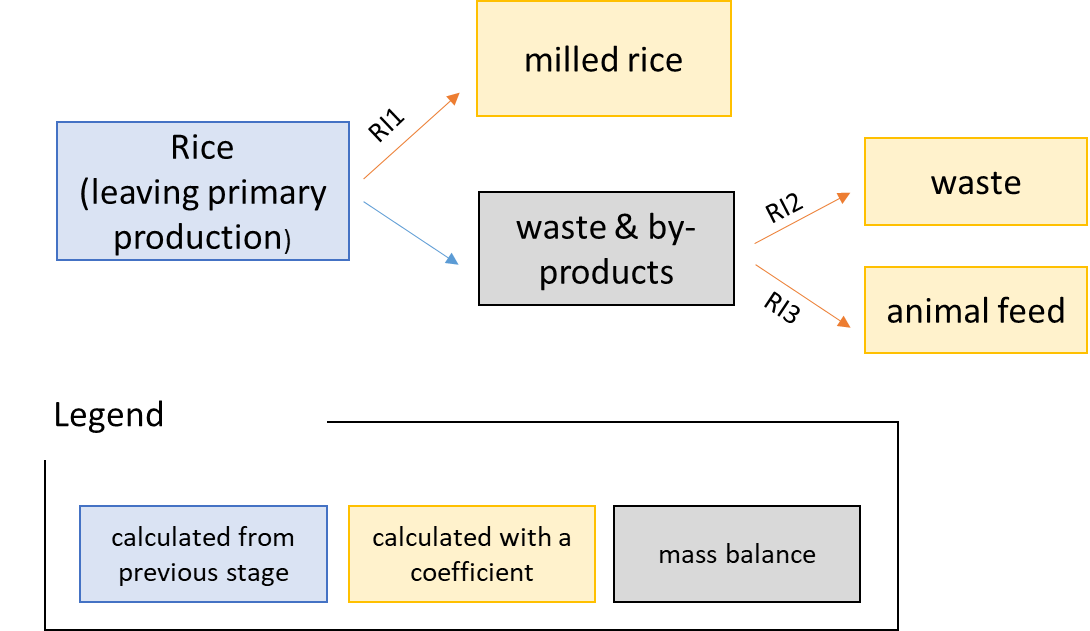


Figure 15: Modelling of rice milling


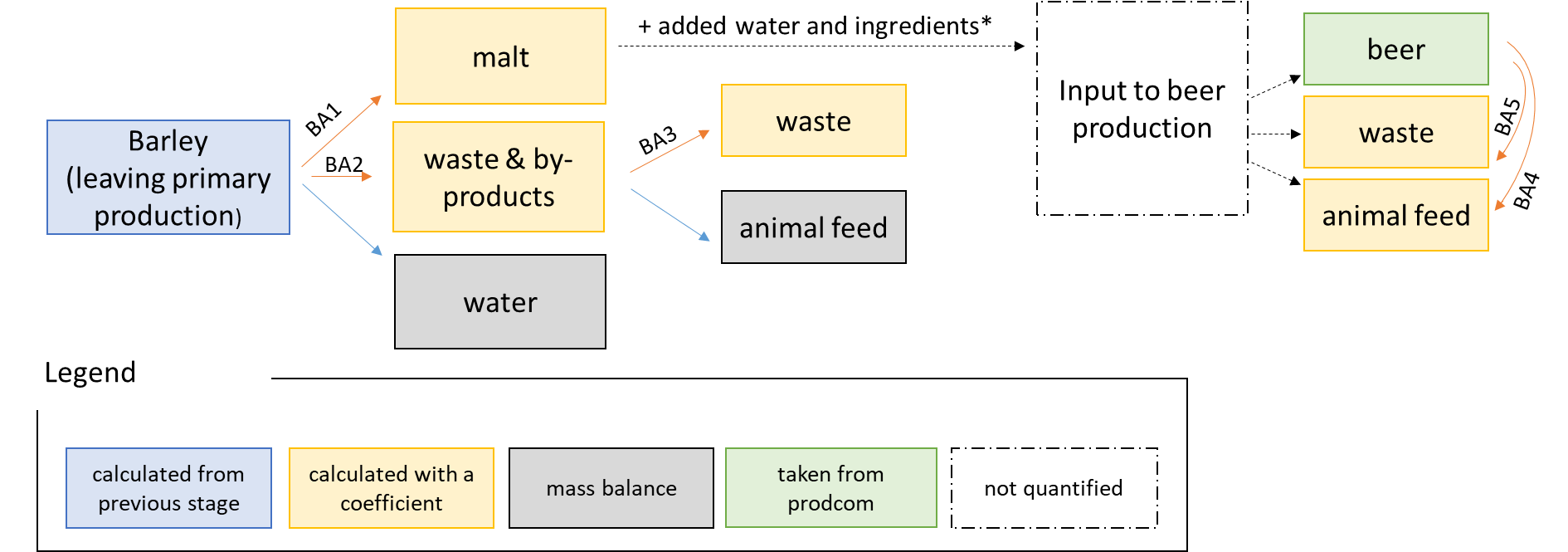


Figure 16: Modelling of barley into beer. * Not quantified.

Finally, the total waste and by-products obtained from the transformation of cereals into processed products are calculated.

To derive the amount of cereal based products entering the distribution phase (as presented in Figure 1) the following procedure is adopted. The amount of flour distributed as such (not used by the food manufacturing sector) is derived from the approach presented above, as illustrated in Figure 12. No information on trade of this product is available, and therefore this element is not considered. For the remaining cereal based products assumed to enter the distribution phase (i.e. bread, pastry, biscuits, pasta, beer, breakfast cereals and rice) information on production and net trade is extracted from Prodcom (see Annex 2, Table 2.5) and added together.

- - 1. Dairy

The processing stage of dairy products is modelled using as input data Eurostat APRO database: specifically considering apro_mk_pobta and apro_mk_farm. These two databases provides for each MS data on whole and skimmed milk utilization to produce dairy products and on the amounts of each product produced in real weight both considering products produced by dairies (apro_mk_pobta) and products produced directly on farms (apro_mk_farm). The following dairy products are included in the model: drinking milk, butter and yellow products, cheese, yogurt, cream, powder and other concentrated milk, casein, buttermilk, milk based drinks, and other fresh products (for more details, see Annex 2, Table A2.7).

It should be noted that in this case the amount of milk leaving primary production (calculated by means of equation 14) is not used to derive the amount entering the P&M stage (conversely to what is reported in Figure 2), which is instead taken directly from the APRO database.

In the model, whey is generated from the production of cheese and caseins taking place at dairies (Figure 17). Whey from cheese production is calculated by using a coefficient taken from (Kemna et al., 2017) multiplied by the amount of milk used to produce cheese, extracted from APRO database. Whey from the production of casein is calculated as the difference between milk (skimmed plus whole) used for caseins production and the amount of caseins produced. From the total whey generated (from caseins and cheese production), a coefficient is applied to calculate the amount that becomes food waste (taken from (Kemna et al., 2017)). Then, whey used for feed is extracted from the APRO database and accounted for as a by-product.

For each dairy product included in the model, the amount produced by dairies is extracted directly from the APRO database.


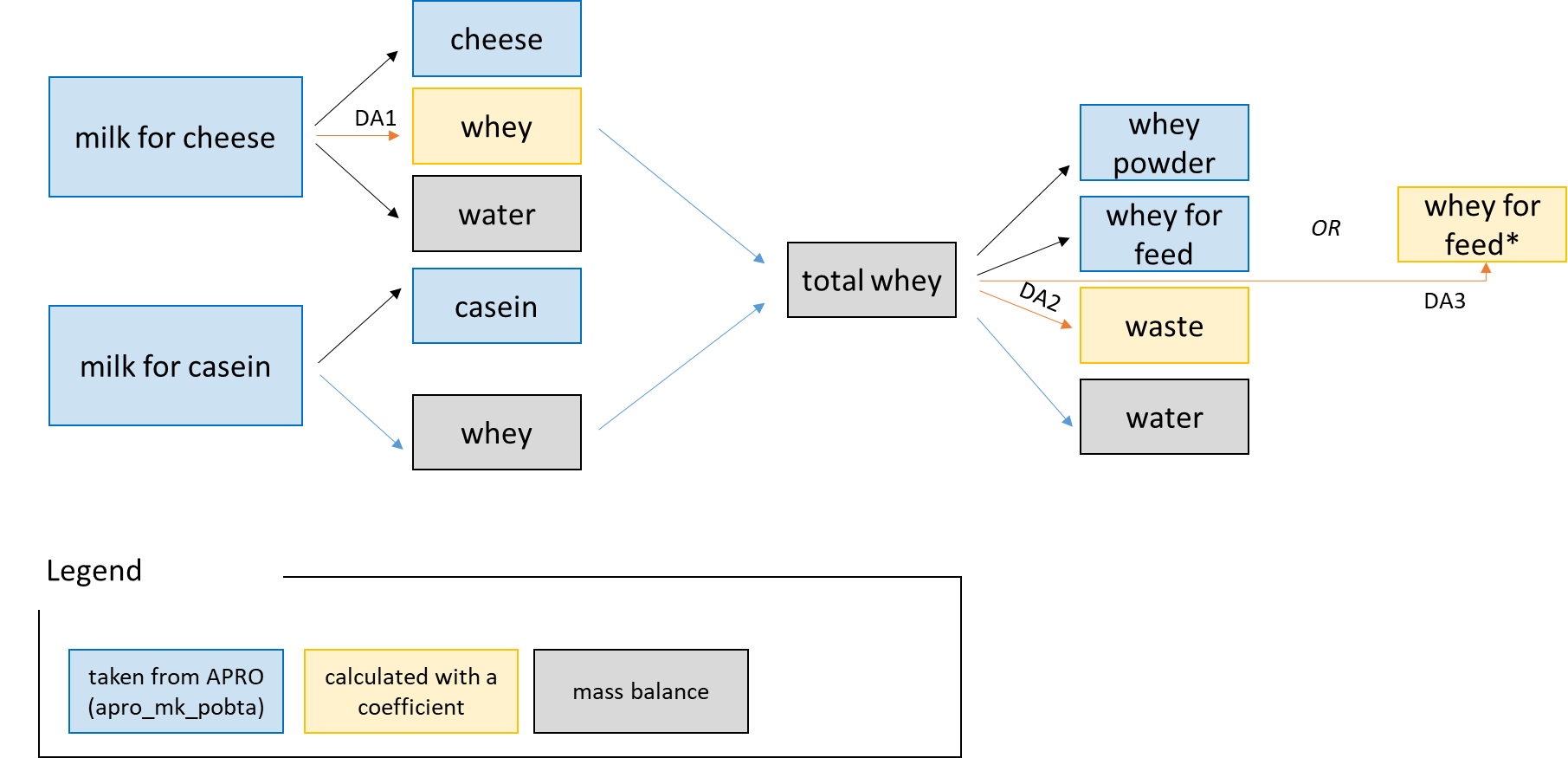


Figure 17: Modelling of milk into cheese and casein products. *in case of missing data in APRO database the whey for feed is calculated from the total whey available by applying a coefficient (DA3).

Regarding the dairy products produced directly at farm level, the following information is extracted from the relevant Eurostat database (apro_mk_farm): the amount of drinking milk, butter, cheese and cream produced on farm. No further assessment is done of waste and by-products generated in the processing of these products, as it is assumed that most residues from these activities would be used as animal feed and they would already be accounted for inside the amount of animal feed extracted from the FBS/CBS, by means of equation 11.

The amount of dairy products entering the distribution phase is derived as the sum of dairy products produced (by dairies and on farm) and net trade of dairy products, extracted from Prodcom database (see Annex 2, Table 2.5).

- - 1. Eggs

The amount of eggs leaving primary production is calculated by means of equation 14. As illustrated by Figure 18, a part of this quantity is distributed fresh while the rest enters the manufacturing stage where it is transformed into processed eggs products (liquid egg, dried egg, and frozen egg) to be sold as such or further used by the food processing industry. The coefficients used to perform this estimation (EG1 and EG2) are provided in Table A1.2 of Annex 1 and derived from (Agra CEAS Consulting, 2008).


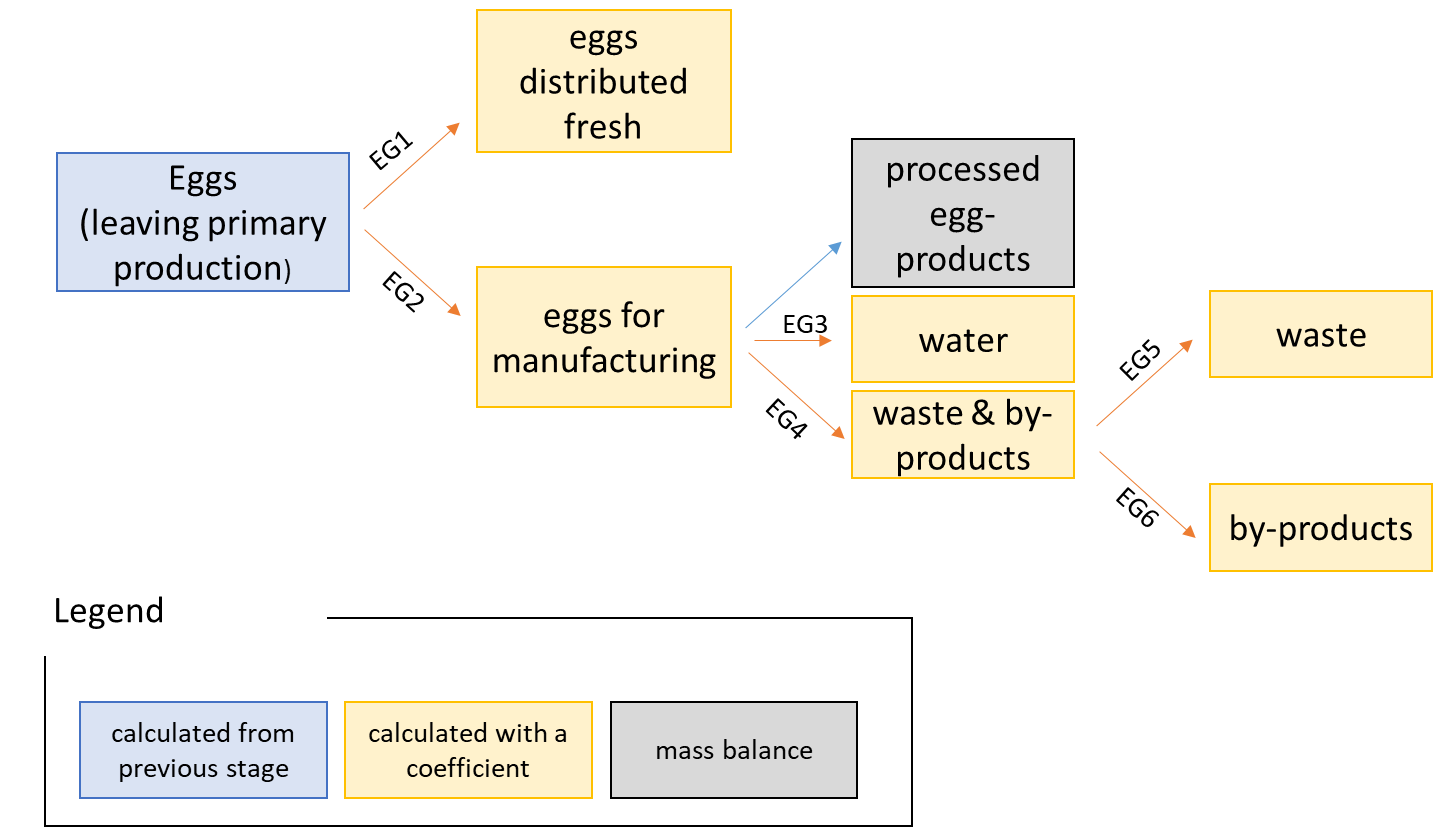


Figure 18: Modelling of processing and manufacturing stage for eggs.

From the quantity entering the manufacturing stage, water evaporated and waste and by-products are calculated by means of coefficients extracted from (Kemna et al., 2017; Rimestad et al., 2017; ROLAND, 1988) (reported in Annex 1, Table A1.2). Hence, the total weight of processed eggs products is calculated through a mass balance, by subtracting from the amount of eggs entering the processing stage, waste, by-products and water leaving the system. This quantity is added to the amount of eggs distributed fresh and to the net trade of processed egg products (extracted from Prodcom, see Annex 2, Table 2.5) to calculate the total amount of eggs and egg products entering the distribution phase (as illustrated in Figure 1).

This is a coarse approximation of what happens in reality, as both fresh and processed egg products are used by the food industry to be incorporated in other processed products (e.g. pasta, biscuits, cakes). Therefore, the flow of egg and egg products entering the distribution phase as such is expected to be lower than the quantity estimated by the model. Nevertheless, it is out of the scope of this project to capture the full complexity of this part of the food supply chain, as this is expected not to affect excessively the estimation of food waste amounts.

- - 1. Meat

The modelling of the processing stage of meat is divided into two sub-stages: slaughtering (presented in Figure 19) and further processing (presented in Figure 20 for all species excluded poultry and Figure 21 for poultry). An overview of the full model of the meat food group is provided in Figure 3. The amount of meat entering the slaughtering stage (live weight) is calculated as explained in Section 1.1.2 (equation 18). From this quantity the waste and by products generated at slaughtering are calculated by means of coefficients that provide the share of CAT1, CAT2 and CAT3 residues generated when slaughtering bovine, sheep, pigs and poultry, as taken from (Laraia et al., 2001), and reported in Annex 1, Table A1.2. Then, wasted amounts and by-products (i.e. residues used for the productions of biodiesel, fertilizers, feed, pet food) are derived using the coefficients reported in Annex 1, retrieved from Ferronato et al., 2020).


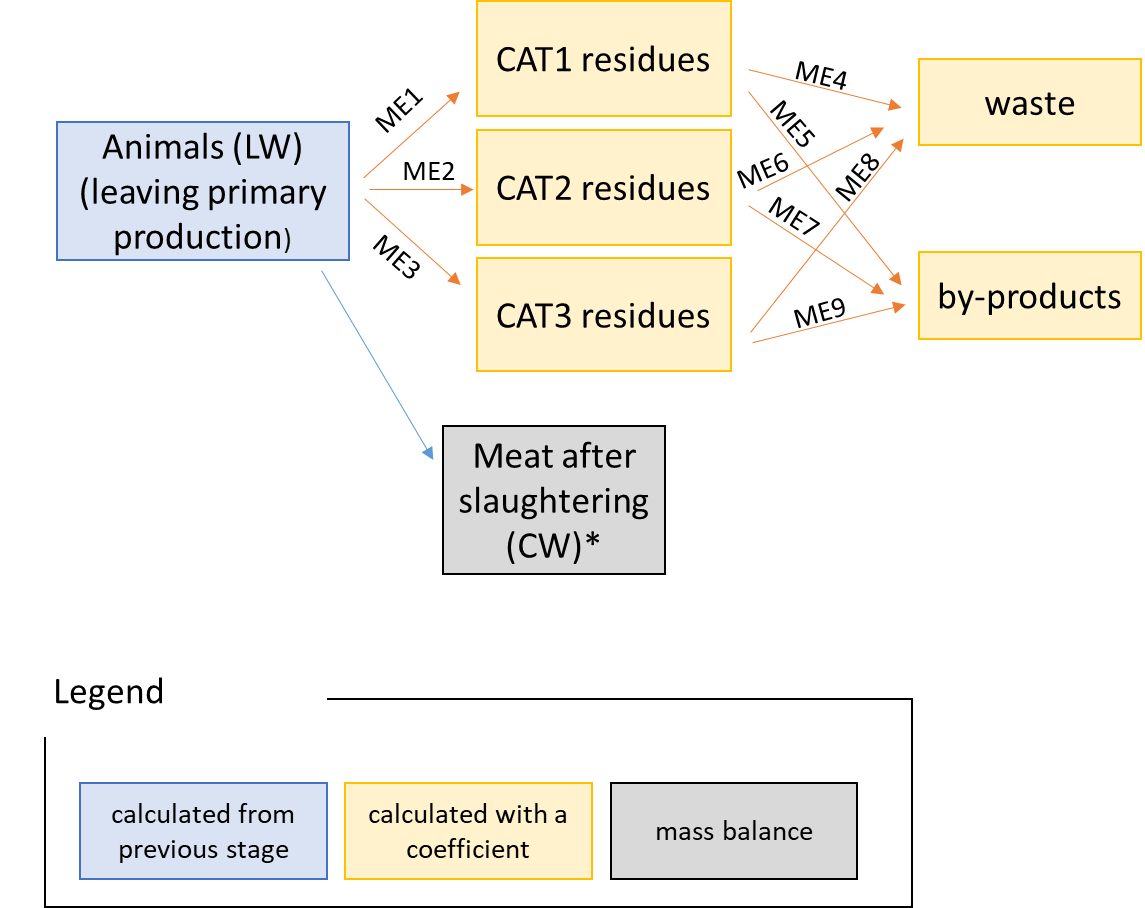


Figure 19: Modelling of meat, all species, slaughtering sub-stage. *This is not linked to the following step (processing) as the amount of meat after slaughtering is taken directly from CBS/FBS. CW: carcass weight

The meat available after slaughtering in each country is calculated from FAOSTAT FBS/CBS and FAOSTAT trade, by means of equation 22 for the following meat products: bovine meat, poultry, pig meat, animal fats, edible offals, other meat (see Annex 2 Table A2.2 for the list of items extracted from the two databases). At this point, meat is expressed in terms of carcass weight^[[5]](#footnote-5)^.

Meat (CW) after slaughtering = P + S + I – E - Other uses* [22]

Where:

P = production (taken from CBS/FBS)

S = stock variation (taken from CBS/FBS)

I = imports (taken from FAO trade)

E = exports (taken from FAO trade)

The term Other uses* is calculated from the value of “Other uses” reported in the FBS/CBS as explained in Section 1.1.1, following equations [3], [10] and [13].


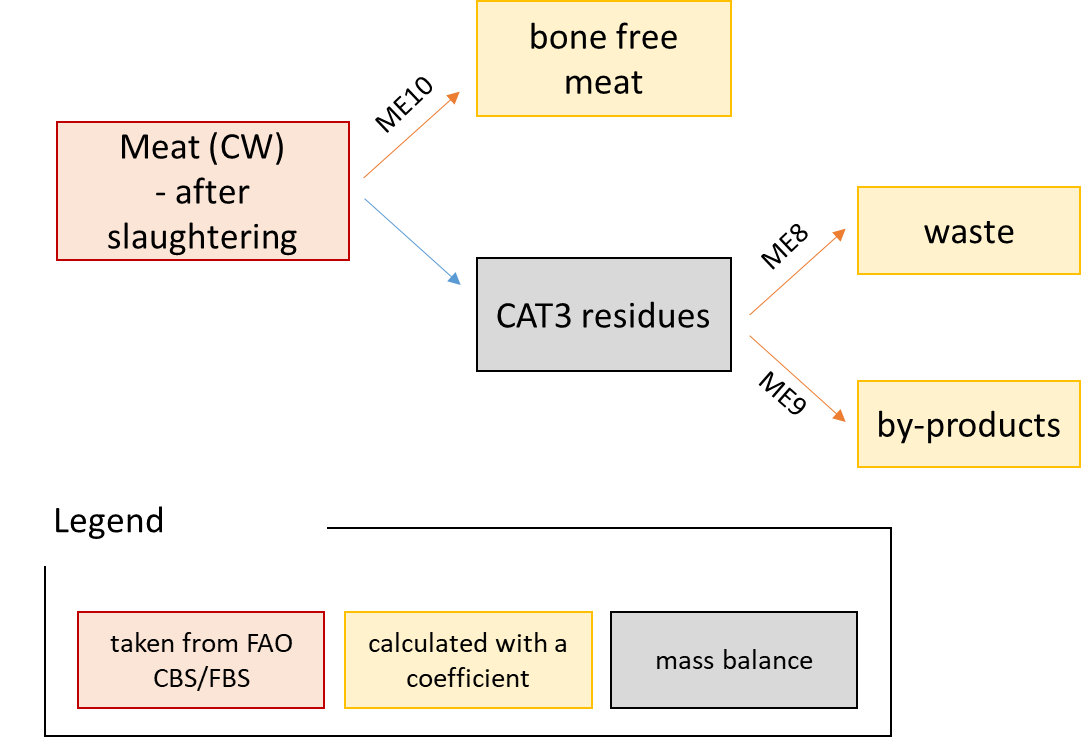


Figure 20: Modelling of the processing stage for bovine, sheep and goat, and pork meat

For all species excluded poultry, as illustrated in Figure 20, the bone-free-meat (BFM) equivalent is calculated, by multiplying the amount of meat available after slaughtering with a coefficient reporting the ratio between BFM and carcass weight for the relative species (ME10). To assign a coefficient to the item “other meat”, the coefficient for sheep was used as a proxy. Instead, for both edible offals and animal fats, a coefficient equal to 1 was used (i.e. assuming the amounts reported in the CBS of these two items are entirely edible). These coefficients are taken from (Ferronato et al., 2020) and reported in Annex 1, table A1.2.


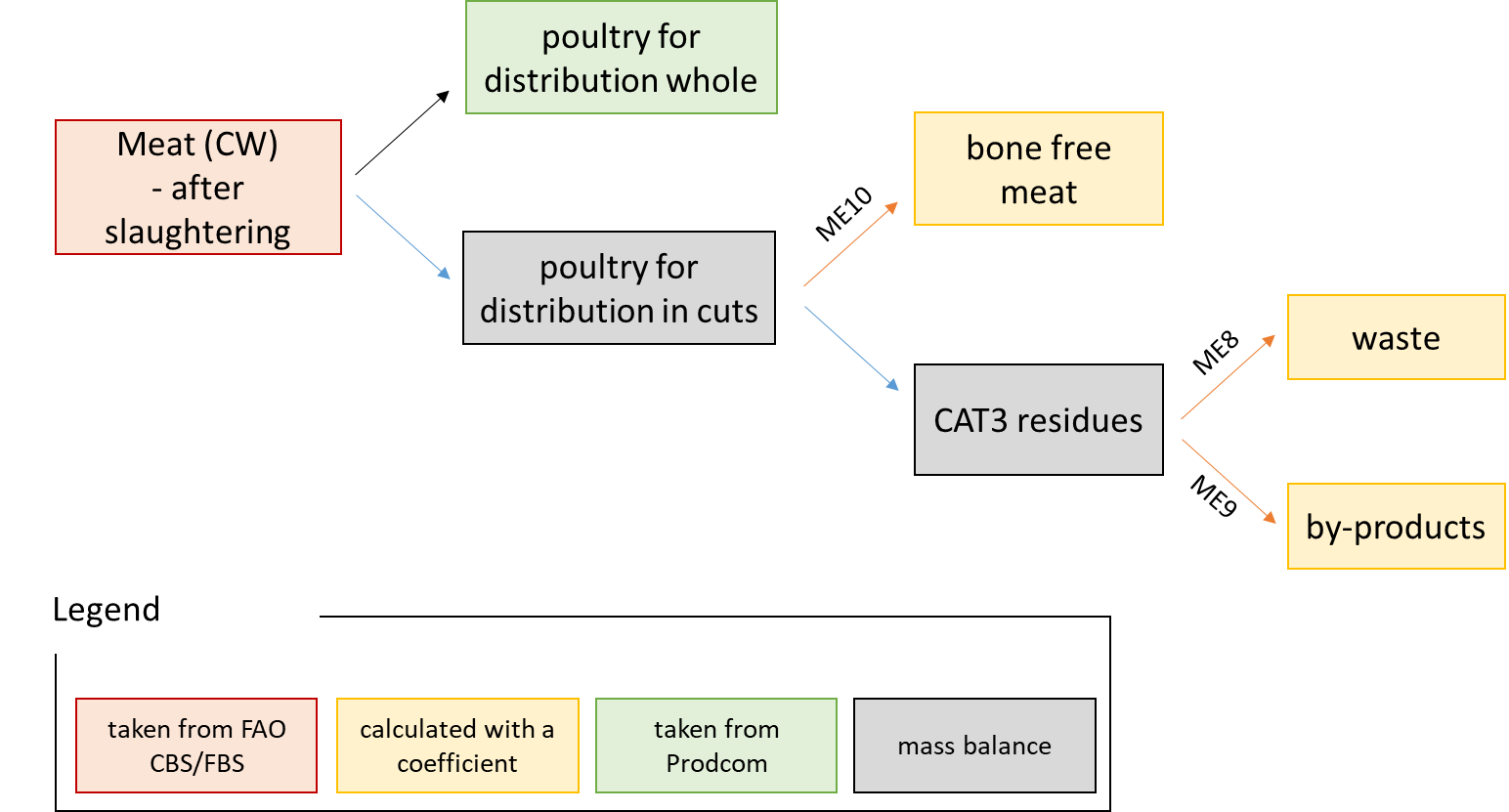


Figure 21: Modelling of the processing stage for poultry meat

In the case of poultry, an additional step is necessary to account for the portion of poultry which is distributed and sold as whole (and therefore should not be converted into BFM at this stage). The amount of poultry distributed as whole is taken from Prodcom (see Annex 2, Table 2.5), and the amount distributed in cuts is calculated by means of mass balance. The latter, is then converted into BFM using the coefficient ME10 specific for poultry, retrieved from (Ferronato et al., 2020).

The residues generated at this stage are then obtained by subtracting from the meat expressed in carcass weight the resulting bone-free-meat. All of these residues are assumed to be CAT3, according to (Ferronato et al., 2020). The same coefficients presented for the other species are used to derive the amount of waste and by-products generated.

Finally, the total meat products entering the distribution stage (as illustrated in Figure 3) are calculated as the sum of: BFM equivalent for each meat product considered (including the portion of poultry meat distributed in cuts), poultry distributed as whole, and net trade of processed meat products (taken from COMEXT, see Annex 2, Table A2.8).

- - 1. Fish

As mentioned in Section 1.1.2, based on statistical data available it is not possible to estimate the flow of fish entering the processing stage. Therefore, unlike the other food groups, the amount of waste and by-products generated at this stage is not calculated from the amount of fish entering this stage, but through an alternative approach here presented, and the P&M stage is modelled in isolation from the remaining stages (as shown in Figure 3).

Firstly, the produced amount of total fish products is taken from EUMOFA for the EU and each MS (see Annex 2, Table 2.9).

According to (Jackson and Newton, 2016), in the EU fish processing activities taking place between 2009 and 2013 generated on average 0.67 Mt of food waste and 1.5 Mt of fish meal and fish oil (classified as by-products) per year. In the same period of time, the average amount of processed fish products produced in the EU was equal to 3.74 Mt. Based on this, two coefficients were calculated and are provided in Annex 1, Table A1.2. The first, FI1, is equal to the ratio between food waste generated and the amount of processed fish products produced in the EU, while the second, FI2, is equal to the ratio between by-products generated and the amount of processed fish products produced in the EU. These two coefficients are then used to calculate food waste and by-products generated by each MS based on the produced amount of fish products, as presented in Figure 22.


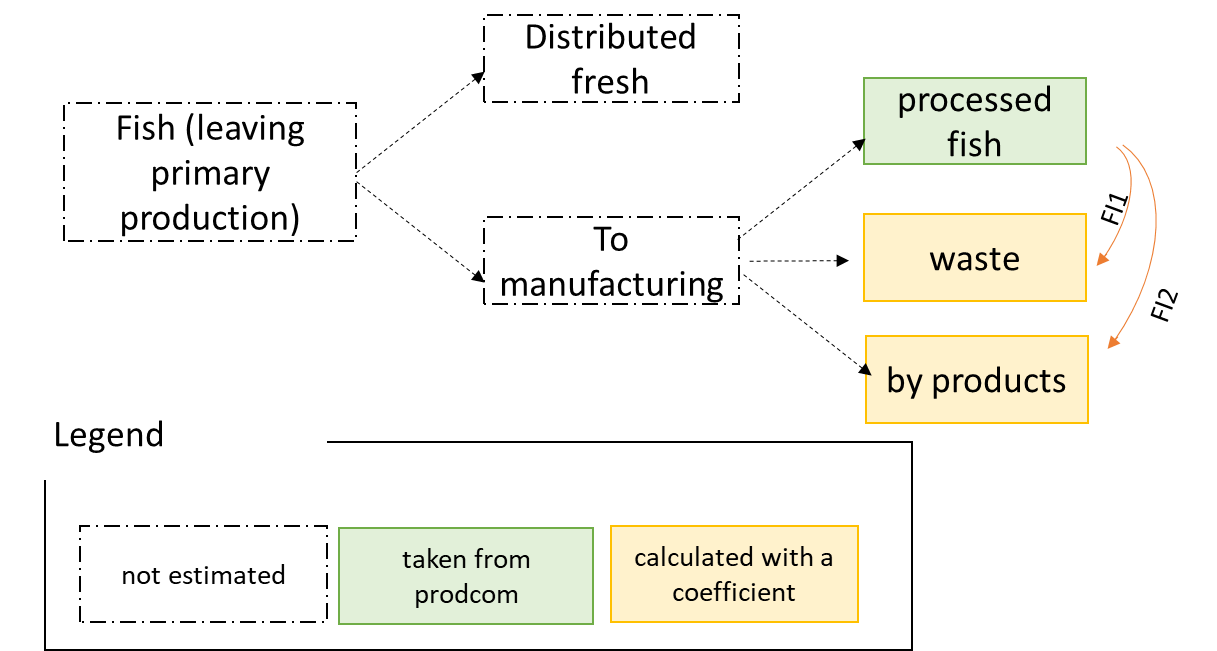


Figure 22: Modelling of the P&M stage of fish

The amount of fish entering the distribution phase is calculated as detailed in section 1.1.2 based on FAOSTAT data and expressed in live weight equivalent. The consequence of this assumption is that food waste generated from the distribution and consumption stage of fish might be overestimated as the mass of fish entering these stages in the model is larger than the real mass of fish. This is because part of the inedible components that are discarded at the processing stage (and accounted for as food waste and by-products at this stage) would be considered to enter the distribution phase due to the methodological choice of assessing the flow of fish in live weight equivalent.

- 1. Retail and distribution (R&D), consumption in household, and consumption in food services

The modelling of the remaining three stages of the supply chain (i.e. distribution and retail, consumption in household, and consumption in food services) is illustrated by Figure 23.

The amount of food entering the R&D stage is calculated for each food group, as presented in Section 1.2. Then, by means of product-specific food waste coefficients, food waste generated at this stage is calculated, and the food entering the consumption stage is obtained through a mass balance.

This quantity is then divided between the food consumed in household and in food services, by means of coefficients. When possible, such coefficients are product and/or country specific, in all other cases, average coefficients are used (coefficients identified with RDC02 – household- and RDC03 – food services - in Annex 1, Table A1.3). Food waste generated at consumption stage is then calculated using food waste coefficients that are specific of the product and of the FSC stage (different coefficients are used for household and food services). The eaten amount of each food group is then calculated by means of mass balances, by subtracting food waste generated from the quantities entering the consumption stage.

Furthermore, coefficients providing the edible waste are applied to derive the amount of food waste that is edible, and by means of mass balances the inedible food waste. All coefficients used, taken from the food waste literature, are provided in Annex 1, Table A1.3.


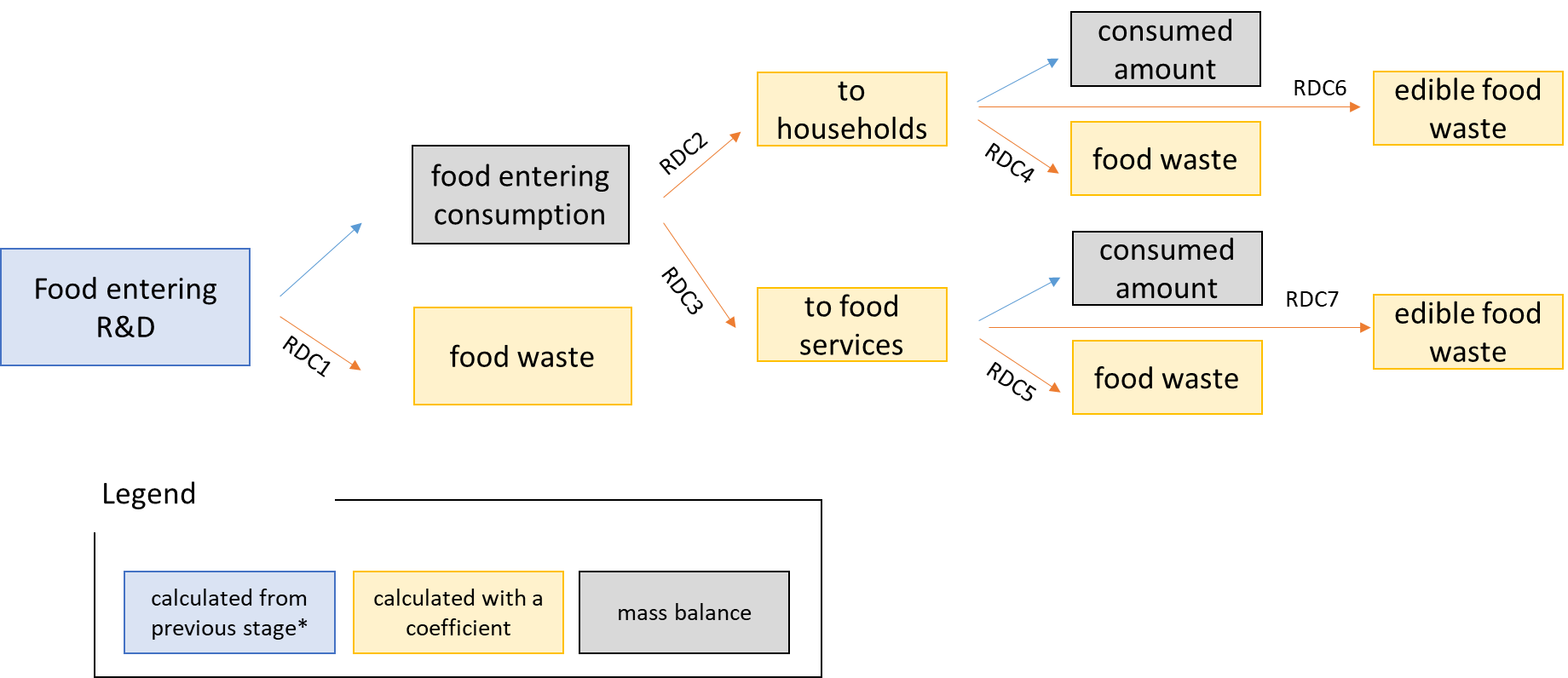


Figure 23: Modelling of retail and distribution and consumption stages. *This calculation varies across food groups, for details see Section 1.1.

## Annexes

**Annex 1. Coefficients used in the model (v1.0) and respective data sources**

**Annex 2. Sources of data used in the model (v1.0) and respective classification**

## References

ADEME, 2016. Pertes et gaspillages alimentaires: l’etat des lieux et leur gestion par etapes de la chaine alimentaire. Available at: <https://www.ademe.fr/sites/default/files/assets/documents/pertes-et-gaspillages-alimentaires-201605-rapport.pdf> (Accessed December 2020)

Agra CEAS Consulting, 2008. The EU egg production sector, Final report for Euro Group.

Caldeira, C., De Laurentiis, V., Corrado, S., van Holsteijn, F., Sala, S., 2019. Quantification of food waste per product group along the food supply chain in the European Union: a mass flow analysis. Resour. Conserv. Recycl. 149, 479–488. https://doi.org/10.1016/j.resconrec.2019.06.011

Consejería de Agricultura y Pesca, 2015. Evaluación de la producción y usos de los subproductos de las agroindustrias del olivar en Andalucía. Sevilla, Spain. Available at : <https://www.juntadeandalucia.es/agriculturaypesca/observatorio/servlet/FrontController?action=RecordContent&table=11031&element=1585171&> (Accessed December 2020)

Courtonne, J.Y., Alapetite, J., Longaretti, P.Y., Dupré, D., Prados, E., 2015. Downscaling material flow analysis: The case of the cereal supply chain in France. Ecol. Econ. https://doi.org/10.1016/j.ecolecon.2015.07.007

De Laurentiis, V., Corrado, S., Sala, S., 2018. Quantifying household waste of fresh fruit and vegetables in the EU. Waste Manag. 77, 238–251. https://doi.org/10.1016/J.WASMAN.2018.04.001

EUMOFA, 2018. The EU Fish Market. EU fish Mark. https://doi.org/10.2771/442971

EUPPA, 2020. Email communication with JRC.

European Parliament and Council, 2002. Regulation (EC) No 178/2002 of the European Parliament and of the Council of 28 January 2002 laying down the general principles and requirements of food law, establishing the European Food Safety Authority and laying down procedures in matters of food safety.

European Parliament and of the Council, 2013. Regulation (EU) No 1380/2013 of the European Parliament and of the Council of 11 December 2013 on the Common Fisheries Policy, amending Council Regulations (EC) No 1954/2003 and (EC) No 1224/2009 and repealing Council Regulations (EC) No 2371/2002 and (EC) No 639/2004 and Council Decision 2004/585/EC.

FAO, 2001. Food balance sheets. A handbook. Food and Agriculture Organization of the United Nations, Rome, Italy.

FAO, 2003. Technical conversion factors for agricultural commodities. Food and Agriculture Organization of the United Nations, Rome, Italy. Avaliable at: http://www.fao.org/fileadmin/templates/ess/documents/methodology/tcf.pdf

Ferronato, G., Corrado, S., De Laurentiis, V., Sala, S., n.d. Holistic assessment of the meat production and consumption system: material flow analysis and assessment of environmental impacts of the Italian supply chain. Submitted.

Freshfel Europe, 2019. Freshfel Fruit and Vegetable Production, Trade, Supply & Consumption Monitor in the EU-28. Brussels: Freshfel Europe.

GSARS, 2017. Guidelines for the Compilation of Food Balance Sheets. Global Strategy Improving Agricultural and Rural Statistics, Rome, Italy.

Hartikainen, H., Mogensen, L., Svanes, E., Franke, U., 2018. Food waste quantification in primary production – The Nordic countries as a case study. Waste Manag. 71. https://doi.org/10.1016/j.wasman.2017.10.026

Jackson, A., Newton, R., 2016. Project To Model The Use of Fisheries By-Products in The Production of Marine Ingredients, with Special Reference The Omega 3 Fatty Acids EPA and DHA, INSTITUTE OF AQUACULTURE, UNIVERSITY OF STIRLING AND IFFO, THE MARINE INGREDIENTS ORGANISATION. Available at: <https://www.iffo.com/system/files/downloads/PROJECT%20TO%20MODEL%20OF%20FISHERIES%20BY%20PRODUCTS%20JULY%202016_IFFO.pdf> (Accessed December 2020)

Kemna, R., van Holsteijn, F., Lee, P., Sims, E., 2017. Optimal food storage conditions in refrigeration appliances. Preparatory/review study on Commission Regulation (EC) No. 643/2009 and Commission Delegated Regulation (EU) No. 1060/2010 – complementary research on Optimal food storage conditions in refrigerators.

Laraia, R., Riva, G., & Squitieri, G. (2001). I rifiuti del comparto agroalimentare. Studio Di Settore. Rapporto ANPA, 148. Available at: https://www.isprambiente.gov.it/contentfiles/00003800/3854-rapporti-01-11.pdf

Rimestad, A.H., Løken, E.B., Nordbotten, A., 2017. The Norwegian food composition table and the database for nutrient calculations at the Institute for Nutrition Research. Available at: <http://www.matportalen.no/verktoy/the_norwegian_food_composition_table/> (Accessed: October 2020)

Roland, D.A., 1988. Research Note: Egg Shell Problems: Estimates of Incidence and Economic Impact. Poult. Sci. 67. https://doi.org/10.3382/ps.0671801

Roth, M., Jekle, M., Becker, T., 2019. Opportunities for upcycling cereal byproducts with special focus on Distiller’s grains. Trends Food Sci. Technol. https://doi.org/10.1016/j.tifs.2019.07.041

Transport and Environment. (2017). 10 Things You Didn’t Know About EU Biofuels. October 2017, 1–13. Available at: <https://www.transportenvironment.org/publications/reality-check-10-things-you-didn%E2%80%99t-know-about-eu-biofuels-policy> (Accessed December 2020)

1. Beyond the different temporal coverage, the main difference between the two databases are that the new food balance sheets provide additional information compared to the commodity balance sheets (e.g. per capita food supply quantity, protein supply quantity), which are not used by the model. For the purpose of this model the two databases are therefore considered equivalent. [↑](#footnote-ref-1)
2. Rice is considered at this stage as “paddy equivalent”. As in the FBS it is only reported as “milled equivalent”, this was converted into “paddy equivalent” by dividing it by a coefficient equal to 0.6 taken from (FAO, 2003) (see Annex 2, Table A2-1). [↑](#footnote-ref-2)
3. <http://gleami.org/> [↑](#footnote-ref-3)
4. https://www.eumofa.eu/web/eumofa [↑](#footnote-ref-4)
5. The definition of carcass weight varies across species. For more details see: <https://ec.europa.eu/eurostat/statistics-explained/index.php/Glossary:Carcass_weight> [↑](#footnote-ref-5)
